# Dopamine: a parallel pathway for the modulation of spinal locomotor networks

**DOI:** 10.3389/fncir.2014.00055

**Published:** 2014-06-16

**Authors:** Simon A. Sharples, Kathrin Koblinger, Jennifer M. Humphreys, Patrick J. Whelan

**Affiliations:** ^1^Hotchkiss Brain Institute, University of CalgaryCalgary, AB, Canada; ^2^Department of Comparative Biology and Experimental Medicine, University of CalgaryCalgary, AB, Canada; ^3^Department of Physiology and Pharmacology, University of CalgaryCalgary, AB, Canada; ^4^Department of Clinical Neurosciences, University of CalgaryCalgary, AB, Canada

**Keywords:** dopamine, monoamines, central pattern generator, locomotion, spinal cord

## Abstract

The spinal cord contains networks of neurons that can produce locomotor patterns. To readily respond to environmental conditions, these networks must be flexible yet at the same time robust. Neuromodulators play a key role in contributing to network flexibility in a variety of invertebrate and vertebrate networks. For example, neuromodulators contribute to altering intrinsic properties and synaptic weights that, in extreme cases, can lead to neurons switching between networks. Here we focus on the role of dopamine in the control of stepping networks in the spinal cord. We first review the role of dopamine in modulating rhythmic activity in the stomatogastric ganglion (STG) and the leech, since work from these preparations provides a foundation to understand its role in vertebrate systems. We then move to a discussion of dopamine’s role in modulation of swimming in aquatic species such as the larval xenopus, lamprey and zebrafish. The control of terrestrial walking in vertebrates by dopamine is less studied and we review current evidence in mammals with a focus on rodent species. We discuss data suggesting that the source of dopamine within the spinal cord is mainly from the A11 area of the diencephalon, and then turn to a discussion of dopamine’s role in modulating walking patterns from both *in vivo* and *in vitro* preparations. Similar to the descending serotonergic system, the dopaminergic system may serve as a potential target to promote recovery of locomotor function following spinal cord injury (SCI); evidence suggests that dopaminergic agonists can promote recovery of function following SCI. We discuss pharmacogenetic and optogenetic approaches that could be deployed in SCI and their potential tractability. Throughout the review we draw parallels with both noradrenergic and serotonergic modulatory effects on spinal cord networks. In all likelihood, a complementary monoaminergic enhancement strategy should be deployed following SCI.

Neuromodulators are the key ingredient allowing motor networks the flexibility to produce multiple patterns of output. When one considers a task such as stepping, a number of patterns need to be produced to run, walk, hop, go up and down inclines and walk in a circular pattern. While we focus on stepping in this review, neuromodulators contribute to the rhythmic operation of most, if not all, biological networks. A complete understanding of the role of neuromodulators in motor control must take into account the combinatorial effects of the complement of transmitters released both synaptically and extrasynaptically. This is an immense challenge for the field of motor control, especially when small invertebrate motor circuits are compared directly with their larger, less accessible and more complex mammalian cousins. However, in the last decade new genetic tools have become available that allow spinal circuits that compose a motor network to be identified (Goulding, 2009). In addition, the birth of optogenetics and pharmacogenetics has provided advanced tools for activating and inactivating neuromodulatory systems (Shapiro et al., 2012; Aston-Jones and Deisseroth, 2013). Even with these new tools it will still be a challenge to examine and comprehend the combinatorial role of multiple neurotransmitters, but the task is more tractable than it has been historically.

Although many neuropeptides, hormones or monoamines can modulate motor circuits, our review will focus on the role of dopamine. Dopamine plays an important role in the activation and modulation of the motor system across a wide range of invertebrate and vertebrate species (Harris-Warrick et al., 1998; Svensson et al., 2003; Puhl and Mesce, 2008; Miles and Sillar, 2011; Clemens et al., 2012; Lambert et al., 2012). In humans, movement disorders such as Parkinson’s disease and Restless Leg Syndrome provide prime examples of what can happen and how debilitating it can be for the individual when the dopaminergic system is compromised. Because of this, a large degree of effort has been focused on understanding the contribution and mechanisms of supraspinal dopamine to motor control. Considerably less attention has been paid towards the descending dopaminergic projections to motor networks in the spinal cord compared to the descending serotonergic and noradrenergic systems. We will discuss and contrast current knowledge of the descending dopaminergic system with the descending serotonergic and noradrenergic system in the control of locomotion in a variety of species, with particular focus on work conducted in the rodent.

## Dopamine’s role in locomotion

Across invertebrate and vertebrate species, dopamine has profound and diverse effects on rhythmically active motor networks. These actions are a result of a complex modulation of intrinsic cellular properties and synaptic connectivity (for review see Harris-Warrick et al., 1998). Remarkably, in some species dopamine demonstrates the ability to reconfigure circuits or networks to generate completely different motor behaviors (Puhl and Mesce, 2008; Crisp et al., 2012; Puhl et al., 2012). More recent studies have highlighted the ability of dopamine to shape a motor network during development, producing a change in motor behaviors from immature to more mature adult-like behaviors (Lambert et al., 2012). Furthermore, dopamine also possesses the capacity to promote developmental and adult motor neurogenesis (Reimer et al., 2013).

Early exploration of the role of catecholamines in locomotor behaviors in the 1960’s determined that L-DOPA could modulate reflex circuits and promote locomotor activity, although at the time it was thought to be of noradrenergic origin (Jankowska et al., 1967a,b; Grillner and Zangger, 1979). For many years, L-DOPA was used to evoke locomotor activity in spinalized cats. It was later demonstrated that L-DOPA could promote air stepping in the neonatal rodent (Sickles et al., 1992; McCrea et al., 1997; McEwen et al., 1997), an effect that is blocked by both noradrenergic (Taylor et al., 1994) and dopaminergic antagonists (Sickles et al., 1992). This work established that catecholamines act on networks that generate locomotion. This work was unclear on the individual effects of dopamine, or the products of dopamine synthesis (noradrenaline) on locomotion and whether they were acting within the spinal cord. Additional studies now suggest that noradrenaline and dopamine both contribute to the control of locomotion in a different way than the predominant spinal serotonergic system that contributes to both evoking and modulating locomotor behavior (Forssberg and Grillner, 1973; Kiehn et al., 1999; Whelan et al., 2000; Jordan et al., 2008; Humphreys and Whelan, 2012).

## Dopamine’s role in rhythmicity: invertebrates

Work from invertebrate species has largely influenced the way that we view modulation of motor systems. For example, the stomatogastric nervous system is a well-defined series of interconnected ganglia that controls the rhythmic filtering and chewing motor patterns of the crab and lobster foregut. The effector ganglion, specifically known as the stomatogastric ganglion (STG), consists of 26–30 neurons that generate a pacemaker-driven higher frequency pyloric rhythm and a conditionally active and lower frequency gastric mill rhythm. Neuromodulation profoundly reconfigures this small circuit, biasing individual neurons to participate in the different motor behaviors. Within even the simplest of nervous systems rhythmic behaviors are not hard-wired, and neuromodulation imbues networks with the flexibility to generate multiple patterns of motor output (Marder, 2012; Gutierrez et al., 2013). Dopamine modulates every aspect of the pyloric circuit including intrinsic membrane properties such as I_A_(Harris-Warrick et al., 1995; Kloppenburg et al., 1999), I_h_ (Peck et al., 2006), I_CAN_ (Kadiri et al., 2011), I_K(V)_ (Gruhn et al., 2005), the strength and dynamics of graded and spike-dependent synaptic transmission (Johnson and Harris-Warrick, 1990; Johnson et al., 2005, 2011; Kvarta et al., 2012) and even properties of the axon spike initiation zone itself (Bucher et al., 2003). It is pertinent to acknowledge that dopamine produces concentration dependent rhythms in both vertebrates and invertebrates (Clemens et al., 2012) and therefore the state of the network can be affected by neuromodulatory tone. Harris-Warrick and Johnson (2010) have reviewed data demonstrating that dopamine can produce opposing effects on multiple conductances in individual neurons of the circuit. This complex modulation depends on the cell type, but the central idea is that dopamine prevents (at least in the STG) runaway modulation of the circuits. Less is known about the role dopamine plays in the gastric mill rhythm. We still do not fully understand the role of dopamine within this circuit; a sobering realization considering the complexity of spinal cord CPG circuits. What we can learn from work on invertebrates is that a neuromodulator can have a strong influence on individual cells, and even differential effects on individual cells in a circuit. However, if we are to understand the overall modulation of motor behavior we need to consider the combinatorial actions of many neuromodulators on network function and not simply one in isolation. There is another factor at play, namely that network output often remain remarkably robust despite neuromodulatory inputs. Furthermore, a change in network output pattern can be elicited in multiple ways through degenerate mechanisms (Gutierrez et al., 2013). This work is an important extension of previous findings showing that a large number of solutions exist even within a simple circuit for the production of a single behavior (Prinz et al., 2004).

Work from the laboratory of Karen Mesce in the medicinal leech (*Hirudo Medicinalis*) illustrates that dopamine has a profound effect on locomotor behaviors, highlighting its ability to bias the network toward a particular motor output or reinforce an ongoing behavior. Specifically, dopamine acts as a command signal to elicit crawling along with suppression of swimming (Puhl and Mesce, 2008; Crisp et al., 2012; Puhl et al., 2012), and is an excellent example of the ability of dopamine to bias locomotor behavior (Crisp and Mesce, 2004). Dopamine has a similar effect in nematodes (e.g., *Caenorhabditis elegans)* where it also acts to bias locomotor activity to a crawl pattern of activity over swimming (Vidal-Gadea et al., 2011). Similar to neuromodulation of the STG, dopamine could be acting on overlapping populations of neurons that are involved in two separate behaviors (Briggman et al., 2005), biasing circuit configuration output toward one output over the other.

## Dopamine’s role in locomotion: aquatic and amphibious species

In the lamprey, dopamine elicits a complex modulatory effect on swimming behavior similar in some respects to rhythmically active motor behaviors of invertebrates. Spinal dopamine is released from a number of sources including small cells located around the central canal that send projections into the CSF of the central canal (Ochi et al., 1979; McPherson and Kemnitz, 1994; Pierre et al., 1997), and also in a more ventrally-located plexus of cells that co-release serotonin and interact with the complex dendritic process of motor neurons (Schotland et al., 1995). There are also descending dopaminergic projections from the hypothalamus that may play a role in modulating spinal networks, but their role with respect to locomotion is not well understood (Barreiro-Iglesias et al., 2008). Therefore, the lamprey exhibits both intrinsic and extrinsic dopaminergic neuromodulation of spinal circuits. Because of the co-release of locally-produced spinal dopamine and serotonin, their effect will be discussed in parallel. When bath applied to the spinal cord *in vitro*, serotonin reduces the frequency and increases the amplitude of locomotor bursting activity in a dose dependant manner (Harris-Warrick and Cohen, 1985), an effect that is mediated by 5-HT_1A_ receptors and is readily reproduced in the freely swimming animal (Kemnitz et al., 1995). These effects are elicited by presynaptic inhibition of descending Muller cells which would result in reduced descending excitation of locomotor circuits (Buchanan and Grillner, 1991; Shupliakov et al., 1995). In addition, serotonin also directly reduces the late afterhyperpolarization (AHP) in motor neurons, lateral interneurons (van Dongen et al., 1986), crossed caudal commissural interneurons and giant interneurons (Wallén et al., 1989). Dopamine appears to have more complex effects, whereby low concentrations (0.1–10 µM) result in an increase in locomotor frequency, higher concentrations (10–100 µM) slow the rhythm, and concentrations as high as 100 µM–1 mM can suppress the rhythm (Harris-Warrick and Cohen, 1985; McPherson and Kemnitz, 1994; Schotland et al., 1995; Svensson et al., 2003). The increase in locomotor frequency can be reproduced in freely swimming animals (Kemnitz et al., 1995) and is believed to be mediated by selective D_2_ receptor mechanisms (McPherson and Kemnitz, 1994). It was initially proposed that this effect was exerted by decreasing the calcium-dependant potassium channel (SK_Ca_) and reducing the late component of the AHP in neurons such as edge cells, dorsal cells and giant interneurons, although these cells are not strong contributors to rhythmogenesis. It is more likely that the influence on rhythm frequency is due to a reduction in inhibitory drive from inhibitory commissural interneurons (Kemnitz, 1997). Concentration dependent dopamine effects are also observed in tadpoles, where effects on mainly D_2_ receptors were observed at low concentrations with D_1_ effects emerging when concentrations of dopamine were increased (Clemens et al., 2012).

The slowing effect of dopamine on locomotor activity at higher concentrations was unclear until recently, but is now believed that it may be acting on convergent mechanisms with that of 5-HT. Both 5-HT_1A_ and D_2_ receptors appear to reduce post-inhibitory rebound (PIR) on inhibitory commissural interneurons that generate left-right alternation of swimming by reducing the calcium conductance through the CaV_1.3_ channel (Hill et al., 2003; Wang et al., 2011). Assuming the organization of a classical half center, the authors suggested that the reduction in PIR would decrease the efficiency of the transition from inhibition to excitation, thus reducing the overall frequency of the alternating pattern.

In contrast to the lamprey, spinal dopamine appears to have a transient effect on swimming in the larval zebra fish with depressive effects at 3 days post fertilization (dpf) becoming less potent by 5 dpf (Thirumalai and Cline, 2008). Further work demonstrates that dopamine contributes to the development and maturation of locomotor networks in the spinal cord (Lambert et al., 2012). The sole source of spinal dopamine in the zebrafish is the Orthopedia (transcription factor, *otp*) neurons of the midbrain forming the dopaminergic diencephalospinal tract (DDT) that send descending projections to the spinal cord (McLean and Sillar, 2004a,b). *Otp* transcription factors are conserved in mammalian species and are expressed in the A11 dopaminergic neurons of the mouse, and analogous areas of the zebrafish, which project to the spinal cord (Ryu et al., 2007). In the zebrafish, it appears that these neurons develop around 3 dpf and act on the D_4_ receptor to reconfigure the locomotor network to generate a more mature form of locomotor behavior; by 4 dpf the actions have switched, from spontaneous swim episodes consisting of infrequent, long duration bursts to frequent and short duration bursting episodes of swimming (Lambert et al., 2012). Such a modification in locomotor behavior is presumably critical for survival, as it would allow the animal to engage in more active locomotor behaviors associated with foraging whereas the immature form is more directed toward the escape from larger predatory fish. More recent investigation of the spinal dopaminergic system in the zebrafish has demonstrated that the D_4a_ receptor also acts on neural progenitor cells via sonic hedgehog signaling to promote motor neuron generation over V2 interneurons during the first 24–48 h post fertilization; an effect that can be recapitulated to promote neuronal regeneration following spinal cord injury (SCI; Reimer et al., 2013). Dopamine does not work alone in shaping the motor network during development. There is evidence that the descending serotonergic system also plays an important role in the development of a mature swimming pattern after the switch occurs at 4 dpf (Brustein et al., 2003). This points to the possibility that dopamine may play a role in promoting the network shift and serotonin may play more of a role in the modulation of a more mature network in the zebrafish. This is consistent with the idea proposed from work in the STG whereby different monoamines likely act simultaneously and rarely on their own.

More is known with respect to the monoaminergic modulation of locomotor networks by serotonin in the *Xenopus* tadpole. In the larval *Xenopus*, the modulatory role of serotonin on fictive swimming activity is to increase burst duration and intensity with no effect on cycle period, thus promoting a stronger swimming pattern (Sillar et al., 1998). This is in contrast to the noradrenergic system which acts to bias the motor output toward a lower frequency and weaker bursting pattern of activity (Sillar et al., 1998). Similar to the lamprey (Wang et al., 2011), both serotonin and noradrenaline modulate locomotor activity through converging mechanisms. However, in the X*enopus* they influence the strength of inhibitory post synaptic potentials evoked by glycinergic commissural interneurons projecting to motor neurons via modulation of presynaptic release of glycine from commissural terminals (McDearmid et al., 1997). More specifically, serotonin reduces release of glycine whereas noradrenaline increases glycine release from these terminals onto motor neurons (McDearmid et al., 1997). In the larval X*enopus* locomotor network, dopamine reduces locomotor activity via D_2_-like mechanisms at low concentrations (1–5 µM) and promotes locomotor activity at higher concentrations (10–50 µM) via D_1_-like mechanisms (Clemens et al., 2012). The precise neuronal populations being acted on are unknown. The development of limbed locomotion in the frog from swimming in the tadpole affords a unique opportunity to study the developmental and potentially evolutionary reconfiguration of the neuronal networks mediating the two different, yet similar, forms of locomotion. In the metamorphosing froglet, with both immature limbs and a tail for swimming still intact, both noradrenaline and serotonin bias locomotor network output controlling either the tail or the developing legs. Specifically, serotonin acts to slow rhythmic tail-associated swimming activity and speeds up the limb-associated activity, whereas noradrenaline will speed up tail-associated swimming activity and slow down limb-associated activity. This provides yet another example of opposing aminergic modulation of distinct spinal locomotor circuits and their functional coupling during amphibian metamorphosis (Rauscent et al., 2009). These are similar to modulatory interactions influencing the pyloric and gastric mill rhythms in the STG (Gutierrez et al., 2013) and expression of swimming and crawling behaviors in the medicinal leech (Crisp and Mesce, 2004; Puhl and Mesce, 2008).

## Biochemical and neuroanatomical aspects of descending dopaminergic systems in mammals

Walking behavior in terrestrial legged mammals recruits up to 80 individual muscles. While the activity of all of them has not been measured during walking, all indications are that each muscle generates a uniquely patterned burst. This adds another layer of complexity to aquatic based species by adding on components of the network that produce not only left-right alternation but also coordination of flexor and extensor muscles within limbs. In mammalian systems, the role of dopamine in rhythmic motor behaviors is less well understood. That said, data collected thus far suggest a modulatory role qualitatively similar to that observed in the lamprey.

## Catecholamine-containing cells

**Figure 1:**
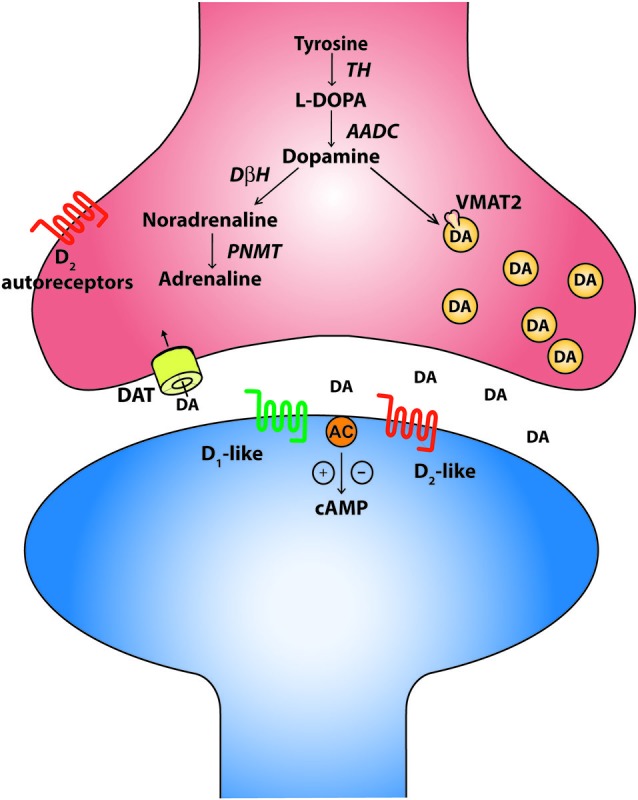
**The canonical catecholaminergic neuron is one that generates dopamine, noradrenaline or adrenaline**. Catecholamines are synthesized from tyrosine through a series of biosynthetic steps progressing from tyrosine hydroxylase (TH) producing L-DOPA, aromatic amino acid decarboxylase (AADC) producing dopamine, dopamine-β-hydroxylase (DBH) producing noradrenaline and phenylethanolamine-N-methyl-transferase (PNMT) producing adrenaline. These neurons also express the vesicular monoaminergic transporter (VMAT2) to pump catecholamines into synaptic vesicles. A canonical dopaminergic neuron will also express dopamine reuptake transporters (DAT) and inhibitory D_2_ autoreceptors to regulate presynaptic release of dopamine. Post synaptic targets of dopamine include the excitatory D_1_-like and inhibitory D_2_-like receptors that act through G-protein second messenger cascades, increasing and decreasing intracellular cAMP levels respectively.

Around the same time that the robust influence of monoamines on long-latency flexor reflex afferents and their relationship to half-center function was being discovered in mammalian motor systems in the early 1960’s (Jankowska et al., 1967a,b; Grillner and Zangger, 1979; Baker et al., 1984), nuclei of serotonin and catecholamine-containing cells were described in the mammalian midbrain and hindbrain. These findings were largely based on approaches that utilized histochemical staining against dopamine and noradrenaline (Carlsson et al., 1962; Dahlström and Fuxe, 1964b), later using more modern immunohistochemical approaches to detect the presence of the enzyme tyrosine-hydroxylase (TH; Hökfelt et al., 1984b). Based on such criteria, these cells were coined aminergic cells, or A-cells, and permitted discrete nuclei to be identified and named A1–A17 (Carlsson et al., 1962; Dahlström and Fuxe, 1964a; Hökfelt et al., 1984a). Advances in immunohistochemical methods were later used to identify cells that express other enzymes involved in the synthesis of catecholamines such as aromatic amino acid decarboxylase (AADC), dopamine-β-hydroxylase (D*β*H) and phenylethanolamine-N-methyl-transferase (PNMT; Figure 1). These developments allowed for further characterization between the types of catecholamine-producing cells. The original view that catecholamine-producing cell types consist of dopaminergic, noradrenergic, and adrenergic classes has been expanded and there is evidence of enzymatic diversity within each class. For example, some TH positive cells lack any enzymes for conversion to traditional catecholamine neurons. Others have a portion, but not all, of the enzymatic machinery. A prime example of these cells are the D-cells which contain AADC but lack TH and are situated around the central canal of the spinal cord (Jaeger et al., 1983). They are similar in location and structure to the dopamine-producing LC cells in the lamprey spinal cord (Ochi et al., 1979; McPherson and Kemnitz, 1994; Pierre et al., 1997) and the AADC^+^/TH^−^ D cells of the zebrafish spinal cord (Chatelin et al., 2001). While the function of these cells is not clear, the bottom line is that catecholaminergic phenotypes are more complex than originally thought. Nevertheless, cells that express TH, AADC and are DβH negative can still provide a good indication that the cells may be dopaminergic whereas the DβH positive cells could be either noradrenergic or adrenergic. Such criteria permitted for the identification of nine major dopaminergic nuclei in the brain (Björklund and Dunnett, 2007). The canonical dopaminergic cell would also express machinery for dopamine release such as a vesicular monoaminergic transporter (VMAT2) to pump dopamine into vesicles, a reuptake transporter like the dopamine transporter (DAT) and a means of regulating release of dopamine such as presynaptic D_2_ autoreceptors (Ugrumov, 2009). It is important to note however that some dopamine neurons differentially express D_2_ autoreceptors. The mechanisms of actions at the soma and terminals may also be different (G protein-coupled inwardly-rectifying potassium channel (GIRK) versus Kv1.2 respectively) and some TH^+^ neurons may not even express them at all (Pappas et al., 2008; Ford, 2014). Therefore, expression of D_2_ autoreceptors may not be a pre-requisite of a canonical dopamine cell, but may be a common feature. In fact, diversity appears the rule rather than the exception for catecholaminergic cell types (Ugrumov, 2009).

## Descending catecholaminergic projections

**Figure 2:**
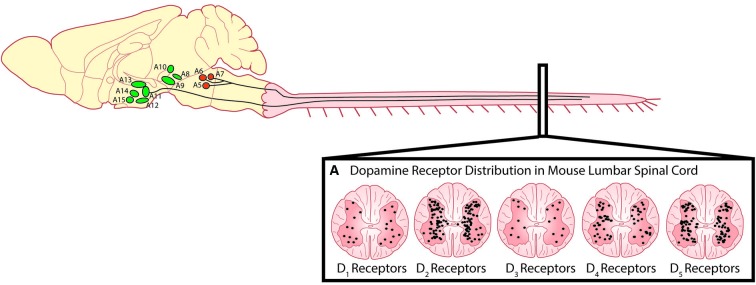
**Descending dopaminergic fibres within the spinal cord originate in the A11.** **(A)** Dopamine acts on all five dopamine receptors that are distributed non-uniformly through the dorsal and ventral horns of the spinal cord (Adapted with permission from Zhu et al., 2007). Descending noradrenergic fibres originating in the A5, A6 and A7 nuclei of the pons innervate the spinal cord. Schematic adapted with permission from Björklund and Dunnett (2007).

The predominant sources of spinal dopamine and noradrenaline in mammals are the descending fibers projecting from the dopaminergic A10 and A11 nuclei of the posterior hypothalamus (Björklund and Skagerberg, 1979; Lindvall et al., 1983; Skagerberg and Lindvall, 1985; Qu et al., 2006; Pappas et al., 2008), the A13 of the dorsal hypothalamus (Blessing and Chalmers, 1979) and the pontine noradrenergic A5, A6 (locus coeruleus) and A7 nuclei (Fritschy and Grzanna, 1990; Clark and Proudfit, 1991; Bruinstroop et al., 2012). While these regions appear to be conserved in many mammalian species including primates and humans (Moore and Bloom, 1979; Barraud et al., 2010), we will focus on these regions with emphasis on rodent anatomy as they are best described in these species. The A11 is a small nucleus in the posterior hypothalamus consisting of approximately 150–300 neurons (Figure 2) and is the primary source of spinal dopamine in mammalian species. A11 neurons are thought to be L-DOPAergic based on the absence of AADC in non-human primates, although the evidence in rodents is mixed (Barraud et al., 2010). This suggests a possibility that L-DOPA release in the spinal cord of the non-human primate could be converted to dopamine by other cells using spinally located AADC such as that present in the D cells near the central canal (Jaeger et al., 1983). It appears that dopaminergic neurons in the A11 go against what a canonical dopaminergic neuron would look like, since A11 neurons lack the reuptake transporter DAT (Lorang et al., 1994; Ciliax et al., 1999) as well as D_2_ auto-receptors (Pappas et al., 2008). The lack of D_2_ autoreceptors would presumably increase release probability for dopamine as well as TH enzyme activity (Kehr et al., 1972; Wolf and Roth, 1990; Benoit-Marand et al., 2001; Ford, 2014). One possibility is that the A11 phenotype serves as a compensatory adaptation given their few numbers in order to prolong the release, and increase synthesis, of dopamine to their targets in the ventral horn.

Although the A11 connectome is not well characterized, it has been shown that the A11 receives inputs from a number of regions including lateral septum, parabrachial nucleus, infralimbic cortex and the bed nucleus of the striata terminalis (Abrahamson and Moore, 2001; Qu et al., 2006). The A11 also receives inputs from the suprachiasmatic nuclei (SCN), suggesting that A11 neurons may be modulated in a circadian pattern (Zhao et al., 2007). There is evidence that this might be the case as the expression of the rate limiting enzyme TH is modulated in a circadian pattern, generating high levels of spinal dopamine during awake periods (Hammar et al., 2004). It has also been suggested that AADC is expressed in a circadian pattern, which may also describe the variable findings to date (Björklund and Dunnett, 2007; Barraud et al., 2010).

The best characterized of the A11 efferent projections are the descending axons that travel through the dorsal longitudinal fasciculus of Schutz, located in the periaqueductal gray, and descend unilaterally through the dorsolateral funiculus into the superficial dorsal horn. A small number of axons also descend in the regions around the central canal and into the ventral horn (Björklund and Skagerberg, 1979; Commissiong et al., 1979; Skagerberg and Lindvall, 1985), with higher densities in the lumbar region relative to the thoracic regions (Pappas et al., 2008). Several examples have been provided by Pappas et al. (2008, 2010), indicating sexual dimorphism of the descending A11 system whereby males have a greater number of dopaminergic neurons and a higher density of descending fibers with no apparent difference in dopamine metabolism (Pappas et al., 2008, 2010). Overall, it has been suggested that this dimorphism, which is androgen-dependant (Pappas et al., 2010), may contribute to the higher female prevalence of restless leg syndrome, a motor disorder associated with impairments in the spinal dopaminergic system (Pappas et al., 2008). To better understand how the A11 exerts its influence on locomotor behavior, the connectivity of the A11 with other locomotor-related regions in the brain needs to be established. In addition to the spinal cord, A11 neurons project collaterals to the dorsal raphe nucleus and the prefrontal cortex (Peyron et al., 1995). It is possible that other targets are likely.

In parallel to A11 projections, noradrenergic fibers of the A5, A6 and A7 travel through the lateral and ventral funiculi and dorsal surface of the dorsal horn. A particularly high density of fibers from the A5 can be found in the lateral region, A6 in the dorsal and ventral regions and A7 mainly in the lateral region. Previously the termination sites of descending noradrenergic projections were controversial (Fritschy and Grzanna, 1990; Clark and Proudfit, 1991). More recent transgenic approaches have revealed that the A5 regions provides the densest innervation of the thoracic sympathetic preganglionic neurons, A6 densest in the dorsal horn at all levels of the spinal cord and the A7 densest in the ventral horn at all levels of the spinal cord (Bruinstroop et al., 2012). Such an organization would suggest that the A5 nucleus would exert more of an influence over autonomic functions, A6 over modulation of sensory input and A7 to motor function. Interestingly, in regards to dopamine innervation of the spinal cord, there is also a clear autonomic innervation of the intermediolateral nucleus of the spinal cord from the A11. Together with noradrenaline this suggests multiple effects of catecholamines on most inputs and outputs of the spinal cord.

Both descending dopaminergic and noradrenergic projections to the spinal cord share similarities in terms of fiber distribution and points of innervation within the gray matter of the spinal cord, but there are differences in terms of fiber density. Dopaminergic fibers appear to develop slower than noradrenergic fibers. Noradrenergic fiber density appears to peak at 14 days after birth with some pruning taking place up to early adulthood. On the other hand dopamine fiber development occurs slowly throughout development in the spinal cord, peaking at early adulthood (Commissiong, 1983). Generally speaking, the descending fibers heavily innervate the dorsal horns where catecholaminergic influence is exerted via diffuse paracrine release, whereas relatively less innervation of the ventral horn is reported, specifically in lamina IX where direct synaptic connections are made on medium to large dendrites with few axo-somatic synapses of motor neurons (Yoshida and Tanaka, 1988; Rajaofetra et al., 1992; Ridet et al., 1992). Much more is known about the nature of synaptic interaction of motor neurons by the descending noradrenergic and serotonergic fibers. Noradrenergic and serotonergic input to motor neuron pools have typically been described as diffuse (Heckman et al., 2008; Johnson and Heckman, 2010); however, recently the nature of synaptic targets was further described for noradrenergic and serotonergic neurons in cat splenius motor neurons (Montague et al., 2013) and appear to be concentrated on small diameter distal dendrites of motor neurons with few located on somal targets. This distribution implies compartmentalization of synaptic contacts, which has also been suggested as playing an important role in regulating the input-output properties of motor neurons (Montague et al., 2013).

## Targets of spinal dopamine

Dopamine receptors are G-protein coupled receptors and are divided into two subfamilies; the D_1_-like receptors (D_1_ and D_5_) and the D_2_-like receptors (D_2_, D_3_, and D_4_). Generally, activation of the D_1_-like receptor subfamily elicits excitatory effects through a stimulatory G-protein (G_sα_) interacting with adenyl cyclase, subsequently increasing intracellular cAMP levels. In contrast, activation of the D_2_- receptor subfamily hyperpolarizes the cell membrane through an inhibitory G-protein (G_iα_) to close calcium channels, open potassium channels and reduce intracellular cAMP levels (Missale et al., 1998). In the rodent, dopamine released from terminals in the lumbar spinal cord can act on all five dopamine receptors (D_1_–D_5_; Figure 2) which are non-uniformly distributed across the transverse lumbar spinal cord (Zhu et al., 2007). Similar to other monoamines, the distribution of receptors is species dependent (Barraud et al., 2010) and their density is likely also developmentally regulated as dopaminergic receptors are in other areas of the brain (Tarazi and Baldessarini, 2000). Little is known in respect of the rostrocaudal distribution but higher levels of dopamine are found in cervical, opposed to lumbar, segments of the spinal cord (Karoum et al., 1981). In the juvenile mouse, D_2_-like receptor subtypes are strongly expressed in lamina I-III of the dorsal horn with the predominant subtype being the D_3_ receptor (Levant and McCarson, 2001). This distribution has been suggested to mediate dopamine’s anti-nociceptive effects. D_1_-like receptors are most strongly expressed in the ventral horn where the motor circuits reside with motor neurons in particular, expressing all five receptor types (Zhu et al., 2007). While *in situ* hybridization techniques have been able to describe the location in the spinal cord where dopamine receptors are expressed, what remains unknown is whether receptors are compartmentalized within different regions of the identified neurons such as is the case for noradrenergic and serotonergic receptors in cat motor neurons (Montague et al., 2013). These factors could contribute to complex modulation of motor neuron input-output properties and network-based effects reflected by dopamine’s complex effects on locomotor activity. More recently, when the non-human primate spinal cord was examined for dopaminergic expression, a different distribution pattern from rodents was observed. Specifically, no evidence for D_1_ mRNA was observed anywhere in the spinal cord, although D_5_ mRNA was found in the dorsal horn. Overall there appears to be a dorsal horn emphasis for dopamine expression in non-human primates compared to rodents (Barraud et al., 2010).

## Functional effects of spinal dopamine on locomotor networks

**Figure 3:**
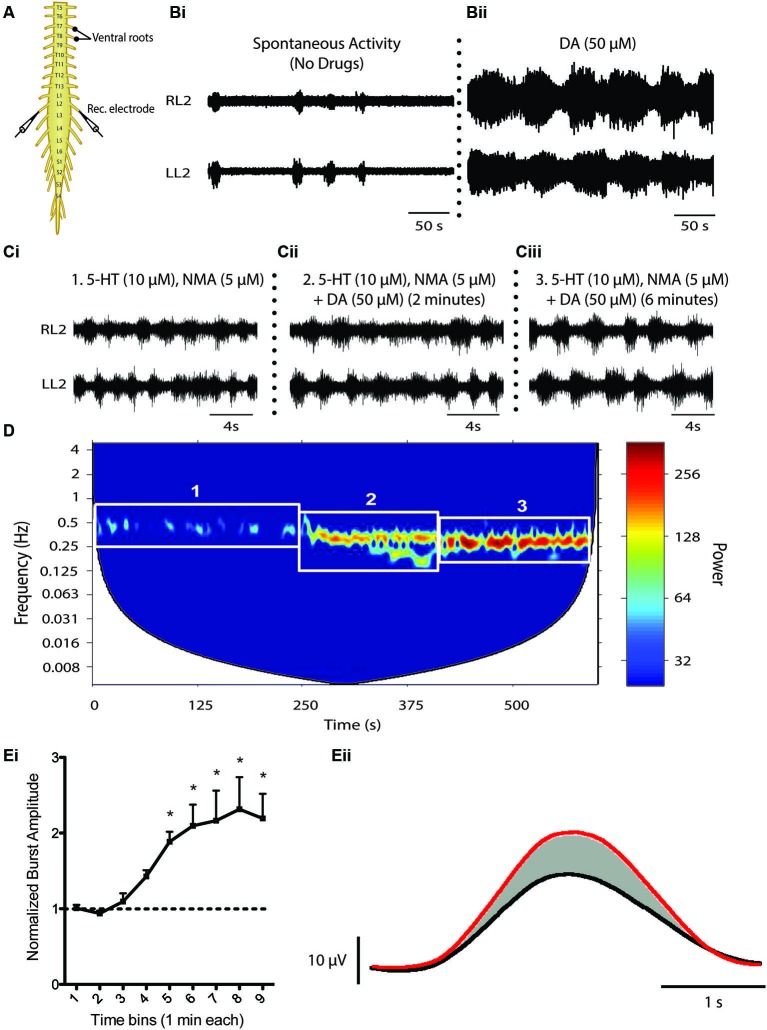
**Dopaminergic modulation of spinal CPG activity**. **(A)** Schematic of an *in vitro* isolated neonatal mouse spinal cord showing ventral root neurograms from the left and right L2 segment. **(B)** Spontaneous activity **(Bi)** is converted to a rhythmic slow non-locomotor rhythmic pattern **(Bii)** following bath application of dopamine (DA). **(C and D)** When DA is bath-applied during ongoing locomotor activity elicited by 5-HT and NMA it stabilizes and reduces the frequency of the rhythm. The spectrogram **(D)** depicts a cross-wavelet analysis of a locomotor rhythm evoked by 5-HT and NMA (box 1) and effect of dopamine (DA) on the pre-existing rhythm (box 2 and 3). Rhythm frequency is displayed on the *y*-axis and rhythm power displayed as warm or cool colors with warmer colors representing higher power or more stable rhythm. Dopamine also increases burst amplitude **(Ei** and **Eii)**. **(Ei)** Graph displays an increase in burst amplitude over a 10 min period immediately following the addition of dopamine and **(Eii)** shows an average L2 neurogram burst from a representative experiment with bursts evoked by 5-HT and NMA (black) and following addition of dopamine (red) (Adapted from Humphreys and Whelan, 2012).

Descending catecholamine release has been observed during locomotion (Gerin et al., 1995; Gerin and Privat, 1998) and both dopamine and noradrenaline are capable of promoting locomotion when exogenously introduced to the intact animal (Barbeau and Rossignol, 1991, 1994; Rossignol et al., 1998). That said, a large amount of what we know about the influence of dopamine on the spinal locomotor networks has been derived from *in vitro* investigations of fictive locomotor activity of the neonatal rodent spinal cord studied in isolation (Whelan et al., 2000; Barrière et al., 2004; Gordon and Whelan, 2006; Humphreys and Whelan, 2012; Christie and Whelan, 2005). Unlike serotonin, which is a potent activator of the locomotor central pattern generator (CPG) network across a variety of mammalian species (Harris-Warrick and Cohen, 1985; Schmidt and Jordan, 2000; Madriaga et al., 2004; Liu and Jordan, 2005; Gabriel et al., 2009), dopamine alone can elicit fictive locomotor activity in the rat (Kiehn and Kjaerulff, 1996; Barrière et al., 2004, but not in the mouse, although D_1_ agonists alone appear to be sufficient for locomotion (Sharples et al., 2013). Bath application of dopamine on its own is sufficient to evoke rhythmic motor activity. However, the low frequency rhythm does not resemble a functional locomotor pattern characterized by left-right and flexor-extensor alternation (Sqalli-Houssaini and Cazalets, 2000; Figure 3). The differential observations with respect to the ability of dopamine and D_1_ agonists to evoke locomotor activity are not yet clear. One possibility is that non-specific binding of dopamine to both D_1_-like and D_2_-like receptor subtypes may act to suppress the full expression of locomotor activity. Noradrenaline does not appear to be capable of eliciting stable locomotor activity in either the isolated rat or mouse spinal cord (Kiehn et al., 1999; Sqalli-Houssaini and Cazalets, 2000). The reasons for the differences between rat and mice in the case of dopamine are unknown but, in the cases where locomotor activity cannot be produced, these two catecholamines generally elicit robust modulatory effects on the lumbar networks (Kiehn et al., 1999; Sqalli-Houssaini and Cazalets, 2000; Gordon and Whelan, 2006; Humphreys and Whelan, 2012).

The behavior of the catecholamines on mammalian motor rhythms should not be surprising in the context of what has been described in the STG where, in all likelihood, neuromodulators influence rhythmic motor behaviors in parallel and rarely in isolation (Marder, 2012). This combinatorial neuromodulation is more evident when ongoing locomotor activity elicited by serotonin and NMA/NMDA is examined where both dopamine and noradrenaline reduce the frequency of the rhythm and increase burst amplitude, resulting in an overall more robust rhythm (Kiehn and Kjaerulff, 1996; Sqalli-Houssaini and Cazalets, 2000; Barrière et al., 2004; Gordon and Whelan, 2006; Humphreys and Whelan, 2012). In other words, these two catecholamines may be promoting ongoing locomotor activity and these qualities are often exploited by incorporating dopamine into the “locomotor cocktail” to elicit robust locomotor activity in investigations that deploy *in vitro* isolated spinal cord models (Whelan et al., 2000).

While neurochemical activation of neonatal locomotor circuits has provided a great amount of insight into the function of locomotor circuits, studies that use models that allow for activation of endogenous neuromodulators are few. In particular, the generation of new models is lacking that allow activation of *in vitro* circuits by direct stimulation of supraspinal nuclei. While these types of studies have been published for serotonin through stimulation of the parapyramidal region of the medulla (Liu and Jordan, 2005), comparable studies have not been performed for catecholamine systems. Furthermore, *in vivo* and developmental studies are required to establish whether dopamine release onto spinal circuits is *necessary* for locomotor activity. This is especially true in neonatal rodents where monoaminergic innervation takes place over 3 weeks following birth (Commissiong, 1983; Bregman, 1987; Rajaofetra et al., 1989).

The receptor mechanisms that mediate the network-based effects of dopamine on locomotor activity are not fully understood. It is likely that dopamine is acting to promote locomotor activity through excitatory influences of D_1_-like receptors in rodents, whereas the slowing effect on fictive locomotor frequency is through inhibitory D_2_-like receptor mechanisms. Indeed, there is consistent evidence across adult and neonatal preparations that the D_1_-like receptor subfamily promotes locomotor activity (Barrière et al., 2004; Lapointe and Guertin, 2008; Lapointe et al., 2009). In contrast, much less is known regarding the role of D_2_-like receptors in the control of locomotion (Barrière et al., 2004) despite their presence in the ventral horn (Zhu et al., 2007). Activation of the D_2_-like receptor subfamily suppresses recurrent excitatory feedback to the locomotor network, most likely via presynaptic mechanisms (Maitra et al., 1993; Humphreys and Whelan, 2012), but the functional role in the modulation of rhythmically active motor circuits in the mammalian spinal cord remains elusive. Further dissection of these receptor mechanisms requires examination of the dopaminergic influence of individual spinal reflex circuits and known components of the spinal locomotor network. Dopamine does elicit potent effects on spinal sensorimotor reflex circuits and has been an area of interest in the context of restless leg syndrome (Carp and Anderson, 1982; Jensen and Yaksh, 1984; Tamae et al., 2005; Barriere et al., 2008; Keeler et al., 2012). D_3_ receptor suppression of primary afferent input to the dorsal horn has been suggested to mediate dopaminergic suppression of the monosynaptic reflex (Hammar et al., 2004) and has been implicated with restless leg syndrome given that D_3_ receptor agonists exert potent therapeutic effects at alleviating motor hyperactivity (Manconi et al., 2011). D_2_-mediated inhibition of primary afferents and post synaptic neurons in the substantia gelatinosa (Tamae et al., 2005) have been suggested to be anti-nociceptive acting at the dorsal horn; an influence that would work in parallel to excitation of motor circuits to promote motor function. What is not well understood is how dopamine may modulate proprioceptive inputs to spinal cord locomotor circuits. In work by Jankowska on the cat, noradrenergic and serotonergic modulation is complex and consists of both excitatory and inhibitory components onto commissural interneurons (Hammar et al., 2004). Similar heterogeneity of dopamine actions is likely but remains underexplored. What is known is that L-DOPA, whether exerting its effect through dopaminergic or noradrenergic systems, modulates afferent reflex circuits by shutting down short latency reflexes and opening up long latency reflexes (Jankowska et al., 1967a,b). This classic work by Anders Lundberg and colleagues showed systemic injection of L-DOPA could reveal an interneuronal network that could result in flexor-extensor alternating movements when flexor and extensor afferents were stimulated simultaneously. This was taken as cellular evidence for Graham-Brown’s half-center concept (Graham-Brown, 1911; Stuart and Hultborn, 2008).

**Figure 4:**
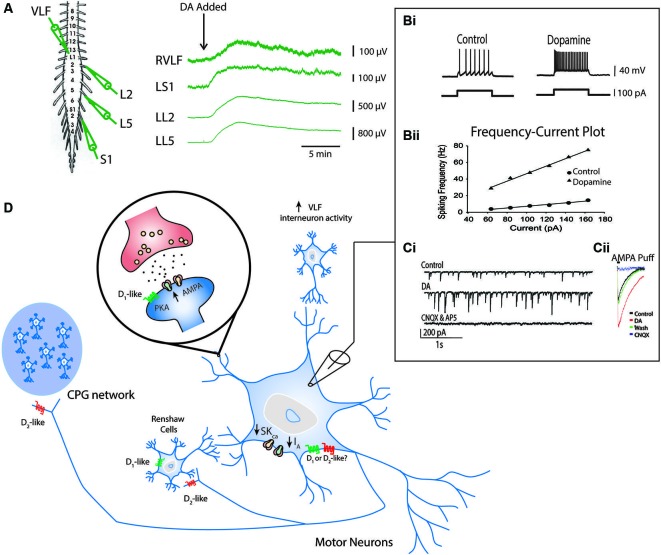
**Known effects of dopamine on cellular components of the locomotor network.** Ventral root neurograms from the L2, L5 and S1 segments and ventrolateral funiculus (VLF) of a spinal cord isolation preparation with fast synaptic transmission blocked (CNQX, AP5, PTX and Strychnine) indicates that dopamine increases the excitability of motor neurons projecting through ventral roots and interneurons projecting through the VLF (A) (Adapted from Han et al., 2007). Intracellular recordings from motor neurons show that dopamine increases motor neuron excitability indicated by an increase in the slope of the frequency-current relationship **(Bi** and **Bii)**. This effect is in part mediated by a reduction in I_A_ and SK_Ca_ conductances. Dopamine also increases AMPA conductances **(Ci** and **Cii)** via a D_1_-like receptor mechanisms (Adapted with permission from Han and Whelan, 2009). Dopamine reduces recurrent excitatory feedback to the locomotor CPG (Humphreys and Whelan, 2012) and Renshaw cells via D_2_-like receptor mechanisms (Maitra et al., 1993). A summary of all these effects are depicted in panel **(D)**.

Dopamine has also been shown to modulate synaptic connectivity and intrinsic properties of some known components of the locomotor network (Han et al., 2007; Han and Whelan, 2009). The ability of dopamine to promote ongoing locomotor activity may be due to combined effects exerted on motor neurons, premotor interneurons and Hb9-expressing interneurons that participate at all levels of the spinal locomotor network (Figure 4; Han et al., 2007; Han and Whelan, 2009). In the neonatal mouse, dopamine acts to depolarize motor neurons as well as premotor interneurons that project through the ventrolateral funiculus (Han et al., 2007 Figure 4A). It appears that dopamine is a necessary component to evoke stable rhythmic bursting activity in classes of genetically identified spinal interneurons (Hb9 cells; Hinckley et al., 2005; Wilson et al., 2005) when introduced with 5-HT and NMDA but is not sufficient to do so on its own (Han et al., 2007); however, other studies suggest that NMDA alone is sufficient (Masino et al., 2012). Dopamine also increases the excitability of motor neurons by reducing the potassium currents I_A_ and SK_ca_, but the receptor mechanisms mediating this effect are not known (Figure 4; Han et al., 2007). In addition, dopamine increases synaptic AMPA currents onto motor neurons via D_1_-like receptor-PKA mechanisms and is analogous to findings in embryonic chick motor neurons mediated by increases in kainite-gated channels (Smith et al., 1995). Together, the combination of increased intrinsic excitability and excitatory synaptic input to motor neurons facilitate motor output; however, it remains unclear why dopamine alone might not be sufficient to evoke locomotor like activity. While these studies are instructive they have only begun to scratch the surface of the function of catecholamines *in vivo*.

## Targeting monoaminergic systems post SCI to promote recovery

Motor impairment is particularly prominent following SCI when the influence from descending serotonergic, noradrenergic and dopaminergic inputs to the lumbar cord are lost or compromised. A number of approaches have been tested experimentally and clinically to promote recovery of motor function following injury and include promoting regeneration of damaged tracts (Hellal et al., 2011), reducing inflammation and subsequent secondary injury (Rowland et al., 2008; Kwon et al., 2011), and promoting plasticity within the spinal motor networks (Boulenguez and Vinay, 2009; Fong et al., 2009; Rossignol and Frigon, 2011). Because monoamines are potent activators of motor networks, they may serve as a suitable target for exciting and promoting recovery following an insult (Rémy-Néris et al., 1999).

Direct application of dopamine or noradrenaline is potentially a useful approach however both would need to be applied intrathecally to spinal segments. For these reasons either agonists or antagonists of catecholamine receptors that can cross the blood brain barrier have been investigated. In chronic spinalized cats, administration of an α_2_ agonist, clonidine, was able to elicit walking in acute spinal cats and promote recovery of function in chronic spinal animals (Forssberg and Grillner, 1973; Barbeau and Rossignol, 1987; Chau et al., 1998), but had the opposite effect in spinalized rats (Musienko et al., 2011). In spinalized mice, locomotor movements were elicited following administration of agonists affecting the D_1_ receptor system (Lapointe et al., 2009), and recent work demonstrates that administration of the D_2_ agonist quinpirole can stabilize gait and facilitate flexion. From these studies we can conclude that dopaminergic agonists potentiate stepping with D_1_ agonists in particular boosting extensor activity and weight-bearing support (Lapointe et al., 2009; Musienko et al., 2011). These data support findings of generalized excitation of motor neurons (Han et al., 2007; Han and Whelan, 2009), and stabilization of motor patterns with dopamine using *in vitro* mouse (Jiang et al., 1999; Whelan et al., 2000; Madriaga et al., 2004; Humphreys and Whelan, 2012) or rat (Barrière et al., 2004) preparations. Courtine and colleagues (Musienko et al., 2011) have recently shown that combinations of monoamines including agonists for 5-HT_1A_, 5-HT_2A_, D_1_ and antagonists for NA (α_2_) were particularly successful in producing locomotion that resembled normal stepping in spinalized rats, supported by data from *in vitro* preparations (Madriaga et al., 2004). A cautionary note is that tuning of these monoaminergic combinations will likely need to be performed before translation to humans in a non-human primate where the receptor expression resembles humans (Barraud et al., 2010). A final note on this topic is that there is evidence that monoamine receptors (5-HT_2C_) are constitutively expressed for months following a SCI in rodents and upregulation of these receptors may contribute to functional improvement in locomotion following injury (Murray et al., 2010). It is not known whether catecholaminergic receptors could contribute in a similar manner but dopamine D_5_ receptors do show high constitutive activity (Demchyshyn et al., 2000). D_5_ receptors are known to be constitutively active in the rat subthalamic nucleus after 6-OHDA treatment of the medial forebrain bundle. D_5_ receptors are expressed in the spinal cord and this remains a possibility for intervention (Chetrit et al., 2013). In this regard, an early report of increased dopamine receptor dependent adenyl cyclase following SCI may be relevant (Gentleman et al., 1981).

Another approach is using L-DOPA that is converted to dopamine and noradrenaline in catecholamine axon terminals and within D cells in the spinal cord. Even though descending catecholamine axons tend to degenerate following injury, D-cells may compensate by upregulating AADC (Jaeger et al., 1983; Li et al., 2014). In spinalized mice, administration of L-DOPA and buspirone (dopamine and 5-HT_1_ partial agonist) combined with apomorphine reliably elicited locomotor activity in mice with a complete spinal transection (Guertin et al., 2011). Work in decerebrate rats suggests that L-DOPA activation of locomotion is dopamine receptor dependent (Sickles et al., 1992; McCrea et al., 1997). In terms of translation, Maric et al. (2008) showed that oral administration of L-DOPA did not produce any observable change in locomotor recovery following SCI. It is not clear why L-DOPA may have proven ineffective but it is likely that a combination of multiple catecholaminergic agonists will need to be developed for humans in combination with other therapies. In this regard, the use of embryonic cells from the dopaminergic ventral tegmental region may also be a useful strategy, similar to approaches that have used embryonic noradrenergic locus coeruleus (Commissiong, 1984) or serotonergic raphe cells implanted into the injury site (Majczyński et al., 2005; Boido et al., 2009).

**Figure 5:**
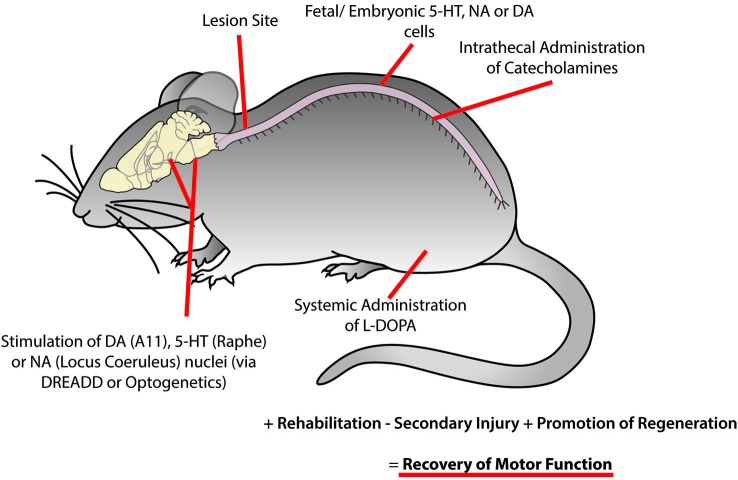
**Recovery of motor function following spinal cord injury may be facilitated using combinatorial therapies to promote plasticity within motor networks**. This could be accomplished by targeting monoaminergic systems through the activation of descending brain nuclei using optogenetic or pharmacogenetic approaches, implantation of fetal or embryonic cells from the dopaminergic ventral tegmental area, noradrenergic locus coeruleus or serotonergic raphe nucleus caudal to the site of injury, intrathecal injection of catecholamines and systemic injection of L-DOPA. While these approaches may not be optimal in isolation, they may serve as an effective complementary treatment with rehabilitation and other therapies to reduce secondary injury and promote regeneration of damaged descending tracts to promote the recovery of motor function.

It is useful to consider new genetic approaches that target monoaminergic sites. The field of optogenetics has developed methods to target classes of cells such that they can be excited or inhibited with light. This is accomplished by the insertion of light sensitive cation channels (e.g., Channelrhodopsin-2) for activation or proton pumps (e.g., archaerhodopsins) for silencing of neuronal activity that can be expressed based on cell-specific transcription factors or promoter regions (for review see: Aston-Jones and Deisseroth, 2013). The use of pharmacogenetics which make use of engineered receptors that respond to non-endogenous ligands could address difficulties of translation, as optogenetic approaches require light fibers to be implanted and would most likely need to cover multiple cord segments. In addition, these non-endogenous ligands can be introduced intravenously, intraperitoneally or orally (for review see: Lee et al., 2014). The most commonly utilized form of this technology are the designer receptors exclusively activated by designer drugs (DREADDS), which are modified metabotropic muscarinic receptors that respond to the non-endogenous ligand clozapine-N-oxide (CNO). Similar to optogenetic approaches, these receptors can be inserted into specific cell types under transcriptional control of cell-specific genes. Neuronal activity can be facilitated through insertion of the hm3 receptor form or silenced by insertion of the hm4 receptor form (Shapiro et al., 2012) by administration of CNO. This approach is considerably simpler than optogenetics, since hardware need not be installed and the CNO can be administered systemically. Following administration of the CNO, effects can be observed within tens of minutes and can last for hours. The next day the CNO will be metabolized and one can then repeat the experimental protocol. Using this approach one can target supraspinal nuclei containing monoaminergic cells in patients with incomplete SCI or direct activation of spinal circuits (Figure 5). This approach has promise since it allows for the remote activation of targets by administering artificial ligands that bind selectively to the artificial receptors targeted to the monoaminergic nucleus of interest. Other approaches to consider are the use of OptoXRs which activate similar cAMP processes as native α_2_ receptors (Airan et al., 2009). The OptoXR approach is light activated at the moment, but offers the opportunity to selectively upregulate α_2_ receptors in motor neurons for example. This technique would directly target second messenger pathways in targeted cells. In all likelihood, a combination of supraspinal plus spinal activation and inactivation strategies will need to be deployed to achieve optimal results. In most cases mentioned the tools used combine Cre-driver mice lines coupled with floxxed viral vectors. However, if translation is a consideration then it should be noted that delivery of DREADDs, opsins, and optoXRs can be delivered using a viral vector with a cell specific synthetic promoter. For example, viral vectors with a TH synthetic promoter have been designed to trace dopamine neuron pathways (Oh et al., 2009). This proof-of-principle shows that this approach could be used in different species including, over the long-term, humans.

## Conclusions

This review has focused on the role of dopamine in modulating locomotor centers in the spinal cord. Our knowledge of dopamine’s contribution to monoaminergic locomotor drive in mammals is in its infancy. However, parallels to other vertebrate systems, such as the lamprey, clearly exist.

Tools to specifically target dopaminergic and other monoaminergic descending populations now exist. Data from behaving animals suggests that while monoamines generally act to increase tone, they can act to promote specific patterns on their own. While the temptation is to examine each monoaminergic system in isolation, it will be necessary to examine combinatorial actions to start to understand the state-dependent role of monoamines in the freely moving animal.

## Conflict of interest statement

The authors declare that the research was conducted in the absence of any commercial or financial relationships that could be construed as a potential conflict of interest.

## References

[B1] Abrahamson E. E. Moore R. Y. (2001). The posterior hypothalamic area: chemoarchitecture and afferent connections. Brain Res. 889, 1–22 10.1016/s0006-8993(00)03015-8 11166682

[B2] Airan R. D. Thompson K. R. Fenno L. E. Bernstein H. Deisseroth K. (2009). Temporally precise in vivo control of intracellular signalling. Nature 458, 1025–1029 10.1038/nature07926 19295515

[B3] Aston-Jones G. Deisseroth K. (2013). Recent advances in optogenetics and pharmacogenetics. Brain Res. 1511, 1–5 10.1016/j.brainres.2013.01.026 23422677 PMC3663045

[B4] Baker L. L. Chandler S. H. Goldberg L. J. (1984). L-dopa-induced locomotor-like activity in ankle flexor and extensor nerves of chronic and acute spinal cats. Exp. Neurol. 86, 515–526 10.1016/0014-4886(84)90086-4 6499992

[B5] Barbeau H. Rossignol S. (1987). Recovery of locomotion after chronic spinalization in the adult cat. Brain Res. 412, 84–95 10.1016/0006-8993(87)91442-9 3607464

[B6] Barbeau H. Rossignol S. (1991). Initiation and modulation of the locomotor pattern in the adult chronic spinal cat by noradrenergic, serotonergic and dopaminergic drugs. Brain Res. 546, 250–260 10.1016/0006-8993(91)91489-n 2070262

[B7] Barbeau H. Rossignol S. (1994). Enhancement of locomotor recovery following spinal cord injury. Curr. Opin. Neurobiol. 7, 517–524 10.1097/00019052-199412000-00008 7866583

[B8] Barraud Q. Obeid I. Aubert I. Barrière G. Contamin H. Mcguire S. (2010). Neuroanatomical study of the A11 diencephalospinal pathway in the non-human primate. PLoS One 5:e13306 10.1371/journal.pone.0013306 20967255 PMC2954154

[B9] Barreiro-Iglesias A. Villar-Cerviño V. Anadón R. Rodicio M. C. (2008). Descending brain-spinal cord projections in a primitive vertebrate, the lamprey: cerebrospinal fluid-contacting and dopaminergic neurons. J. Comp. Neurol. 511, 711–723 10.1002/cne.21863 18925562

[B10] Barriere G. Leblond H. Provencher J. Rossignol S. (2008). Prominent role of the spinal central pattern generator in the recovery of locomotion after partial spinal cord injuries. J. Neurosci. 28, 3976–3987 10.1523/jneurosci.5692-07.2008 18400897 PMC6670475

[B11] Barrière G. Mellen N. Cazalets J.-R. (2004). Neuromodulation of the locomotor network by dopamine in the isolated spinal cord of newborn rat. Eur. J. Neurosci. 19, 1325–1335 10.1111/j.1460-9568.2004.03210.x 15016090

[B12] Benoit-Marand M. Borrelli E. Gonon F. (2001). Inhibition of dopamine release via presynaptic D2 receptors: time course and functional characteristics in vivo. J. Neurosci. 21, 9134–9141 11717346 10.1523/JNEUROSCI.21-23-09134.2001 PMC6763925

[B13] Björklund A. Dunnett S. B. (2007). Dopamine neuron systems in the brain: an update. Trends Neurosci. 30, 194–202 10.1016/j.tins.2007.03.006 17408759

[B14] Björklund A. Skagerberg G. (1979). Evidence for a major spinal cord projection from the diencephalic A11 dopamine cell group in the rat using transmitter-specific fluorescent retrograde tracing. Brain Res. 177, 170–175 10.1016/0006-8993(79)90927-2 497819

[B15] Blessing W. W. Chalmers J. P. (1979). Direct projection of catecholamine (presumably dopamine)-containing neurons from hypothalamus to spinal cord. Neurosci. Lett. 11, 35–40 10.1016/0304-3940(79)90052-1 431883

[B16] Boido M. Rupa R. Garbossa D. Fontanella M. Ducati A. Vercelli A. (2009). Embryonic and adult stem cells promote raphespinal axon outgrowth and improve functional outcome following spinal hemisection in mice. Eur. J. Neurosci. 30, 833–846 10.1111/j.1460-9568.2009.06879.x 19712091

[B17] Boulenguez P. Vinay L. (2009). Strategies to restore motor functions after spinal cord injury. Curr. Opin. Neurobiol. 19, 587–600 10.1016/j.conb.2009.10.005 19896827

[B18] Bregman B. S. (1987). Development of serotonin immunoreactivity in the rat spinal cord and its plasticity after neonatal spinal cord lesions. Brain Res. 431, 245–263 10.1016/0165-3806(87)90213-6 3304541

[B140] Briggman K. L. Abarbanel H. D. I. Kristan W. B. (2005). Optical imaging of neuronal populations during decision-making. Science 307, 896–901 10.1126/science.1103736 15705844

[B19] Bruinstroop E. Cano G. Vanderhorst V. G. J. M. Cavalcante J. C. Wirth J. Sena-Esteves M. (2012). Spinal projections of the A5, A6 (locus coeruleus) and A7 noradrenergic cell groups in rats. J. Comp. Neurol. 520, 1985–2001 10.1002/cne.23024 22173709 PMC3508755

[B20] Brustein E. Saint-Amant L. Buss R. R. Chong M. McDearmid J. R. Drapeau P. (2003). Steps during the development of the zebrafish locomotor network. J. Physiol. Paris 97, 77–86 10.1016/j.jphysparis.2003.10.009 14706693

[B21] Buchanan J. T. Grillner S. (1991). 5-Hydroxytryptamine depresses reticulospinal excitatory postsynaptic potentials in motoneurons of the lamprey. Neurosci. Lett. 122, 71–74 10.1016/0304-3940(91)90196-z 1676146

[B141] Bucher D. Thirumalai V. Marder E. (2003). Axonal dopamine receptors activate peripheral spike initiation in a stomatogastric motor neuron. J. Neurosci. 23, 6866–6875 12890781 10.1523/JNEUROSCI.23-17-06866.2003 PMC6740739

[B22] Carlsson A. Falck B. Hillarp N. A. (1962). Cellular localization of brain monoamines. Acta Physiol. Scand. Suppl. 56, 1–28 14018711

[B23] Carp J. S. Anderson R. J. (1982). Dopamine receptor-mediated depression of spinal monosynaptic transmission. Brain Res. 242, 247–254 10.1016/0006-8993(82)90307-9 6126249

[B24] Chatelin S. Wehrlé R. Mercier P. Morello D. Sotelo C. Weber M. J. (2001). Neuronal promoter of human aromatic L-amino acid decarboxylase gene directs transgene expression to the adult floor plate and aminergic nuclei induced by the isthmus. Brain Res. Mol. Brain Res. 97, 149–160 10.1016/s0169-328x(01)00318-7 11750071

[B25] Chau C. Barbeau H. Rossignol S. (1998). Effects of intrathecal alpha1- and alpha2-noradrenergic agonists and norepinephrine on locomotion in chronic spinal cats. J. Neurophysiol. 79, 2941–2963 9636099 10.1152/jn.1998.79.6.2941

[B26] Chetrit J. Taupignon A. Froux L. Morin S. Bouali-Benazzouz R. Naudet F. (2013). Inhibiting subthalamic D5 receptor constitutive activity alleviates abnormal electrical activity and reverses motor impairment in a rat model of Parkinson’s disease. J. Neurosci. 33, 14840–14849 10.1523/JNEUROSCI.0453-13.2013 24027284 PMC6705171

[B142] Christie K. J. Whelan P. J. (2005). Monoaminergic establishment of rostrocaudal gradients of rhythmicity in the neonatal mouse spinal cord. J. Neurophysiol. 94, 1554–1564 10.1152/jn.00299.2005 15829596

[B27] Ciliax B. J. Drash G. W. Staley J. K. Haber S. Mobley C. J. Miller G. W. (1999). Immunocytochemical localization of the dopamine transporter in human brain. J. Comp. Neurol. 409, 38–56 10.1002/(sici)1096-9861(19990621)409:1<38::aid-cne4>3.0.co;2-1 10363710

[B28] Clark F. M. Proudfit H. K. (1991). The projection of locus coeruleus neurons to the spinal cord in the rat determined by anterograde tracing combined with immunocytochemistry. Brain Res. 538, 231–245 10.1016/0006-8993(91)90435-x 2012966

[B29] Clemens S. Belin-Rauscent A. Simmers J. Combes D. (2012). Opposing modulatory effects of D1- and D2-like receptor activation on a spinal central pattern generator. J. Neurophysiol. 107, 2250–2259 10.1152/jn.00366.2011 22262823

[B31] Commissiong J. W. (1983). Development of catecholaminergic nerves in the spinal cord of the rat. Brain Res. 264, 197–208 10.1016/0006-8993(83)90817-x 6850292

[B32] Commissiong J. W. (1984). Fetal locus coeruleus transplanted into the transected spinal cord of the adult rat: some observations and implications. Neuroscience 12, 839–853 10.1016/0306-4522(84)90174-x 6472623

[B33] Commissiong J. W. Gentleman S. Neff N. H. (1979). Spinal cord dopaminergic neurons: evidence for an uncrossed nigrospinal pathway. Neuropharmacology 18, 565–568 10.1016/0028-3908(79)90102-3 481709

[B143] Crisp K. M. Gallagher B. R. Mesce K. A. (2012). Mechanisms contributing to the dopamine induction of crawl-like bursting in leech motoneurons. J. Exp. Biol. 215, 3028–3036 10.1242/jeb.069245 22660774

[B34] Crisp K. M. Mesce K. A. (2004). A cephalic projection neuron involved in locomotion is dye coupled to the dopaminergic neural network in the medicinal leech. J. Exp. Biol. 207, 4535–4542 10.1242/jeb.01315 15579549

[B35] Dahlström A. Fuxe K. (1964a). Evidence for the existence of monoamine-containing neurons in the central nervous system. I. Demonstration of monoamines in the cell bodies of brain stem neurons. Acta Physiol. Scand. Suppl. 232, 1–55 14229500

[B36] Dahlström A. Fuxe K. (1964b). Localization of monoamines in the lower brain stem. Experientia 20, 398–399 10.1007/bf02147990 5856530

[B37] Demchyshyn L. L. McConkey F. Niznik H. B. (2000). Dopamine D5 receptor agonist high affinity and constitutive activity profile conferred by carboxyl-terminal tail sequence. J. Biol. Chem. 275, 23446–23455 10.1074/jbc.m000157200 10807903

[B39] Fong A. J. Roy R. R. Ichiyama R. M. Lavrov I. Courtine G. Gerasimenko Y. (2009). Recovery of control of posture and locomotion after a spinal cord injury: solutions staring us in the face. Prog. Brain Res. 175, 393–418 10.1016/S0079-6123(09)17526-X 19660669 PMC2904312

[B40] Ford C. P. (2014). The role of D2-autoreceptors in regulating dopamine neuron activity and transmission. Neuroscience [Epub ahead of print]. 10.1016/j.neuroscience.2014.01.025 24463000 PMC4108583

[B41] Forssberg H. Grillner S. (1973). The locomotion of the acute spinal cat injected with clonidine i.v. Brain Res. 50, 184–186 10.1016/0006-8993(73)90606-9 4690545

[B42] Fritschy J. M. Grzanna R. (1990). Demonstration of two separate descending noradrenergic pathways to the rat spinal cord: evidence for an intragriseal trajectory of locus coeruleus axons in the superficial layers of the dorsal horn. J. Comp. Neurol. 291, 553–582 10.1002/cne.902910406 2329191

[B43] Gabriel J. P. Mahmood R. Kyriakatos A. Söll I. Hauptmann G. Calabrese R. L. (2009). Serotonergic modulation of locomotion in zebrafish: endogenous release and synaptic mechanisms. J. Neurosci. 29, 10387–10395 10.1523/JNEUROSCI.1978-09.2009 19692613 PMC6665790

[B44] Gentleman S. Parenti M. Commissiong J. W. Neff N. H. (1981). Dopamine-activated adenylate cyclase of spinal cord: supersensitivity following transection of the cord. Brain Res. 210, 271–275 10.1016/0006-8993(81)90900-8 7225811

[B38] Gerin C. Becquet D. Privat A. (1995). Direct evidence for the link between monoaminergic descending pathways and motor activity. I. A study with microdialysis probes implanted in the ventral funiculus of the spinal cord. Brain Res. 704, 191–201 10.1016/0006-8993(95)01111-0 8788914

[B45] Gerin C. Privat A. (1998). Direct evidence for the link between monoaminergic descending pathways and motor activity: II. A study with microdialysis probes implanted in the ventral horn of the spinal cord. Brain Res. 794, 169–173 10.1016/s0006-8993(98)00278-9 9630613

[B46] Gordon I. T. Whelan P. J. (2006). Monoaminergic control of cauda-equina-evoked locomotion in the neonatal mouse spinal cord. J. Neurophysiol. 96, 3122–3129 10.1152/jn.00606.2006 16956991

[B144] Goulding M. (2009). Circuits controlling vertebrate locomotion: moving in a new direction. Nat. Rev. Neurosci. 10, 507–518 10.1038/nrn2608 19543221 PMC2847453

[B47] Graham-Brown T. (1911). The intrinsic factors in the act of progression in the mammal. Proc. R. Soc. Lond. B Biol. Sci. 84, 308–319 10.1098/rspb.1911.0077

[B48] Grillner S. Zangger P. (1979). On the central generation of locomotion in the low spinal cat. Exp. Brain Res. 34, 241–261 10.1007/bf00235671 421750

[B49] Gruhn M. Guckenheimer J. Land B. Harris-Warrick R. M. (2005). Dopamine modulation of two delayed rectifier potassium currents in a small neural network. J. Neurophysiol. 94, 2888–2900 10.1152/jn.00434.2005 16014791

[B50] Guertin P. A. Ung R.-V. Rouleau P. Steuer I. (2011). Effects on locomotion, muscle, bone and blood induced by a combination therapy eliciting weight-bearing stepping in nonassisted spinal cord-transected mice. Neurorehabil. Neural Repair 25, 234–242 10.1177/1545968310378753 20952632

[B51] Gutierrez G. J. O’Leary T. Marder E. (2013). Multiple mechanisms switch an electrically coupled, synaptically inhibited neuron between competing rhythmic oscillators. Neuron 77, 845–858 10.1016/j.neuron.2013.01.016 23473315 PMC3664401

[B30] Hammar I. Bannatyne B. A. Maxwell D. J. Edgley S. A. Jankowska E. (2004). The actions of monoamines and distribution of noradrenergic and serotoninergic contacts on different subpopulations of commissural interneurons in the cat spinal cord. Eur. J. Neurosci. 19, 1305–1316 10.1111/j.1460-9568.2004.03239.x 15016088 PMC1971244

[B52] Han P. Nakanishi S. T. Tran M. A. Whelan P. J. (2007). Dopaminergic modulation of spinal neuronal excitability. J. Neurosci. 27, 13192–13204 10.1523/jneurosci.1279-07.2007 18045913 PMC6673410

[B53] Han P. Whelan P. J. (2009). Modulation of AMPA currents by D(1)-like but not D(2)-like receptors in spinal motoneurons. Neuroscience 158, 1699–1707 10.1016/j.neuroscience.2008.11.040 19110039

[B54] Harris-Warrick R. M. Cohen A. H. (1985). Serotonin modulates the central pattern generator for locomotion in the isolated lamprey spinal cord. J. Exp. Biol. 116, 27–46 4056654 10.1242/jeb.116.1.27

[B145] Harris-Warrick R. M. Coniglio L. M. Barazangi N. Guckenheimer J. Gueron S. (1995). Dopamine modulation of transient potassium current evokes phase shifts in a central pattern generator network. J. Neurosci. 15, 342–358 7823140 10.1523/JNEUROSCI.15-01-00342.1995 PMC6578330

[B146] Harris-Warrick R. M. Johnson B. R. (2010). Checks and balances neuromodulation. Front. Behav. Neurosci. 4, 1–9 10.3389/fnbeh.2010.00047 20700503 PMC2917248

[B147] Harris-Warrick R. M. Johnson B. R. Peck J. H. Kloppenburg P. Ayali A. Skarbinski J. (1998). Distributed effects of dopamine modulation in the crustacean pyloric network. Ann. N. Y. Acad. Sci. 860, 155–167 9928309 10.1111/j.1749-6632.1998.tb09046.x

[B55] Heckman C. J. Hyngstrom A. S. Johnson M. D. (2008). Active properties of motoneurone dendrites: diffuse descending neuromodulation, focused local inhibition. J. Physiol. 586, 1225–1231 10.1113/jphysiol.2007.145078 17947305 PMC2375668

[B56] Hellal F. Hurtado A. Ruschel J. Flynn K. C. Laskowski C. J. Umlauf M. (2011). Microtubule stabilization reduces scarring and causes axon regeneration after spinal cord injury. Science 331, 928–931 10.1126/science.1201148 21273450 PMC3330754

[B57] Hill R. H. Svensson E. Dewael Y. Grillner S. (2003). 5-HT inhibits N-type but not L-type Ca(2+) channels via 5-HT1A receptors in lamprey spinal neurons. Eur. J. Neurosci. 18, 2919–2924 10.1111/j.1460-9568.2003.03051.x 14656287

[B58] Hinckley C. A. Hartley R. Wu L. Todd A. J. Ziskind-Conhaim L. H. (2005). Locomotor-like rhythms in a genetically distinct cluster of interneurons in the mammalian spinal cord. J. Neurophysiol. 93, 1439–1449 10.1152/jn.00647.2004 15496486

[B59] Hökfelt T. Johansson O. Goldstein M. (1984a). Chemical anatomy of the brain. Science 225, 1326–1334 10.1126/science.6147896 6147896

[B60] Hökfelt T. Martensson M. Björklund A. Kleineau S. Goldstein M. (1984b). “Distributional maps of tyrosine-hydroxylase-immunoreactive neurons in the rat brain,” in Handbook of Chemical Neuroanatomy (Classical Transmitters in the CNS, Part 1) (Vol. 2), eds Björklund A. Hökfelt T. (Amsterdam: Elsevier Science), 277–379

[B61] Humphreys J. M. Whelan P. J. (2012). Dopamine exerts activation-dependent modulation of spinal locomotor circuits in the neonatal mouse. J. Neurophysiol. 108, 3370–3381 10.1152/jn.00482.2012 22993259

[B62] Jaeger C. B. Teitelman G. Joh T. H. Albert V. R. Park D. H. Reis D. J. (1983). Some neurons of the rat central nervous system contain aromatic-L-amino-acid decarboxylase but not monoamines. Science 219, 1233–1235 10.1126/science.6131537 6131537

[B63] Jankowska E. Jukes M. G. Lund S. Lundberg A. (1967a). The effect of DOPA on the spinal cord. 5. Reciprocal organization of pathways transmitting excitatory action to alpha motoneurones of flexors and extensors. Acta Physiol. Scand. 70, 369–388 10.1111/j.1748-1716.1967.tb03636.x 4293473

[B64] Jankowska E. Jukes M. G. Lund S. Lundberg A. (1967b). The effect of DOPA on the spinal cord. 6. Half-centre organization of interneurones transmitting effects from the flexor reflex afferents. Acta Physiol. Scand. 70, 389–402 10.1111/j.1748-1716.1967.tb03637.x 4294400

[B65] Jensen T. S. Yaksh T. L. (1984). Effects of an intrathecal dopamine agonist, apomorphine, on thermal and chemical evoked noxious responses in rats. Brain Res. 296, 285–293 10.1016/0006-8993(84)90064-7 6322926

[B66] Jiang Z. Carlin K. P. Brownstone R. M. (1999). An in vitro functionally mature mouse spinal cord preparation for the study of spinal motor networks. Brain Res. 816, 493–499 10.1016/s0006-8993(98)01199-8 9878874

[B149] Johnson B. R. Brown J. M. Kvarta M. D. Lu J. Y. J. Schneider L. R. Nadim F. (2011). Differential modulation of synaptic strength and timing regulate synaptic efficacy in a motor network. J. Neurophysiol. 105, 293–304 10.1152/jn.00809.2010 21047938 PMC3023374

[B148] Johnson B. R. Harris-Warrick R. M. (1990). Aminergic modulation of graded synaptic transmission in the lobster stomatogastric ganglion. J. Neurosci. 10, 2066–2076 2165519 10.1523/JNEUROSCI.10-07-02066.1990 PMC6570377

[B67] Johnson M. D. Heckman C. J. (2010). Interactions between focused synaptic inputs and diffuse neuromodulation in the spinal cord. Ann. N Y Acad. Sci. 1198, 35–41 10.1111/j.1749-6632.2010.05430.x 20536918 PMC3794674

[B150] Johnson B. R. Schneider L. R. Nadim F. Harris-Warrick R. M. (2005). Dopamine modulation of phasing of activity in a rhythmic motor network: contribution of synaptic and intrinsic modulatory actions. J. Neurophysiol. 94, 3101–3111 10.1152/jn.00440.2005 16014790 PMC1262651

[B151] Jordan L. M. Liu J. Hedlund P. B. Akay T. Pearson K. G. (2008). Descending command systems for the initiation of locomotion in mammals. Brain Res. Rev. 57, 183–191 10.1016/j.brainresrev.2007.07.019 17928060

[B152] Kadiri L. R. Kwan A. C. Webb W. W. Harris-Warrick R. M. (2011). Dopamine-induced oscillations of the pyloric pacemaker neuron rely on release of calcium from intracellular stores. J. Neurophysiol. 106, 1288–1298 10.1152/jn.00456.2011 21676929 PMC3174818

[B153] Karoum F. Commissiong J. C. Neff N. H. Wyatt R. J. (1981). Regional differences in catecholamine formation and metabolism in the rat spinal cord. Brain Res. 212, 316–366 10.1016/0006-8993(81)90468-6 7225873

[B68] Keeler B. E. Baran C. A. Brewer K. L. Clemens S. (2012). Increased excitability of spinal pain reflexes and altered frequency-dependent modulation in the dopamine D3-receptor knockout mouse. Exp. Neurol. 238, 273–283 10.1016/j.expneurol.2012.09.002 22995602

[B69] Kehr W. Carlsson A. Lindqvist M. Magnusson T. Atack C. (1972). Evidence for a receptor-mediated feedback control of striatal tyrosine hydroxylase activity. J. Pharm. Pharmacol. 24, 744–747 10.1111/j.2042-7158.1972.tb09104.x 4404084

[B154] Kemnitz C. P. (1997). Dopaminergic modulation of spinal neurons and synaptic potentials in the lamprey spinal cord. J. Neurophysiol. 77, 289–298 9120571 10.1152/jn.1997.77.1.289

[B70] Kemnitz C. P. Strauss T. R. Hosford D. M. Buchanan J. T. (1995). Modulation of swimming in the lamprey, Petromyzon marinus, by serotonergic and dopaminergic drugs. Neurosci. Lett. 201, 115–118 10.1016/0304-3940(95)12147-1 8848231

[B71] Kiehn O. Kjaerulff O. (1996). Spatiotemporal characteristics of 5-HT and dopamine-induced rhythmic hindlimb activity in the in vitro neonatal rat. J. Neurophysiol. 75, 1472–1482 8727391 10.1152/jn.1996.75.4.1472

[B72] Kiehn O. Sillar K. T. Kjaerulff O. McDearmid J. R. (1999). Effects of noradrenaline on locomotor rhythm-generating networks in the isolated neonatal rat spinal cord. J. Neurophysiol. 82, 741–746 10444672 10.1152/jn.1999.82.2.741

[B155] Kloppenburg P. Levini R. M. Harris-Warrick R. M. (1999). Dopamine modulates two potassium currents and inhibits the intrinsic firing properties of an identified motor neuron in a central pattern generator network. J. Neurophysiol. 81, 29–38 9914264 10.1152/jn.1999.81.1.29

[B156] Kvarta M. D. Harris-Warrick R. M. Johnson B. R. (2012). Neuromodulator-evoked synaptic metaplasticity within a central pattern generator network. J. Neurophysiol. 108, 2846–2856 10.1152/jn.00586.2012 22933725 PMC3545119

[B73] Kwon B. K. Casha S. Hurlbert R. J. Yong V. W. (2011). Inflammatory and structural biomarkers in acute traumatic spinal cord injury. Clin. Chem. Lab. Med. 49, 425–433 10.1515/CCLM.2011.068 21175377

[B74] Lambert A. M. Bonkowsky J. L. Masino M. A. (2012). The conserved dopaminergic diencephalospinal tract mediates vertebrate locomotor development in zebrafish larvae. J. Neurosci. 32, 13488–13500 10.1523/jneurosci.1638-12.2012 23015438 PMC3481997

[B75] Lapointe N. P. Guertin P. A. (2008). Synergistic effects of D1/5 and 5-HT1A/7 receptor agonists on locomotor movement induction in complete spinal cord-transected mice. J. Neurophysiol. 100, 160–168 10.1152/jn.90339.2008 18480366

[B76] Lapointe N. P. Rouleau P. Ung R.-V. Guertin P. A. (2009). Specific role of dopamine D1 receptors in spinal network activation and rhythmic movement induction in vertebrates. J. Physiol. 587, 1499–1511 10.1113/jphysiol.2008.166314 19204052 PMC2678221

[B77] Lee H.-M. Giguere P. M. Roth B. L. (2014). DREADDs: novel tools for drug discovery and development. Drug Discov. Today 19, 469–473 10.1016/j.drudis.2013.10.018 24184433 PMC4004703

[B78] Levant B. McCarson K. E. (2001). D(3) dopamine receptors in rat spinal cord: implications for sensory and motor function. Neurosci. Lett. 303, 9–12 10.1016/s0304-3940(01)01692-5 11297811

[B79] Li Y. Li L. Stephens M. J. Zenner D. Murray K. C. Winship I. R. (2014). Synthesis, transport, and metabolism of serotonin formed from exogenously applied 5-HTP after spinal cord injury in rats. J. Neurophysiol. 111, 145–163 10.1152/jn.00508.2013 24068759 PMC3921369

[B80] Lindvall O. Björklund A. Skagerberg G. (1983). Dopamine-containing neurons in the spinal cord: anatomy and some functional aspects. Ann. Neurol. 14, 255–260 10.1002/ana.410140302 6314870

[B81] Liu J. Jordan L. M. (2005). Stimulation of the parapyramidal region of the neonatal rat brain stem produces locomotor-like activity involving spinal 5-HT7 and 5-HT2A receptors. J. Neurophysiol. 94, 1392–1404 10.1152/jn.00136.2005 15872068

[B82] Lorang D. Amara S. G. Simerly R. B. (1994). Cell-type-specific expression of catecholamine transporters in the rat brain. J. Neurosci. 14, 4903–4914 8046459 10.1523/JNEUROSCI.14-08-04903.1994 PMC6577178

[B83] Madriaga M. A. McPhee L. C. Chersa T. Christie K. J. Whelan P. J. (2004). Modulation of locomotor activity by multiple 5-HT and dopaminergic receptor subtypes in the neonatal mouse spinal cord. J. Neurophysiol. 92, 1566–1576 10.1152/jn.01181.2003 15163678

[B84] Maitra K. K. Seth P. Thewissen M. Ross H. G. Ganguly D. K. (1993). Dopaminergic influence on the excitability of antidromically activated Renshaw cells in the lumbar spinal cord of the rat. Acta Physiol. Scand. 148, 101–107 10.1111/j.1748-1716.1993.tb09538.x 8352022

[B85] Majczyński H. Maleszak K. Cabaj A. SŁacuteńska U. (2005). Serotonin-related enhancement of recovery of hind limb motor functions in spinal rats after grafting of embryonic raphe nuclei. J. Neurotrauma 22, 590–604 10.1089/neu.2005.22.590 15892603

[B157] Manconi M. Ferri R. Zucconi M. Clemens S. Giarolli L. Bottasini V. (2011). Preferential D2 or preferential D3 dopamine agonists in restless legs syndrome. Neurology 77, 110–117 10.1212/WNL.0b013e3182242d91 21715702

[B86] Marder E. (2012). Neuromodulation of neuronal circuits: back to the future. Neuron 76, 1–11 10.1016/j.neuron.2012.09.010 23040802 PMC3482119

[B87] Maric O. Zörner B. Dietz V. (2008). Levodopa therapy in incomplete spinal cord injury. J. Neurotrauma 25, 1303–1307 10.1089/neu.2008.0583 19061374

[B88] Masino M. A. Abbinanti M. D. Eian J. Harris-Warrick R. M. (2012). TTX-resistant NMDA receptor-mediated membrane potential oscillations in neonatal mouse Hb9 interneurons. PLoS One 7:e47940 10.1371/journal.pone.0047940 23094101 PMC3475713

[B89] McCrea A. E. Stehouwer D. J. van Hartesveldt C. (1997). Dopamine D1 and D2 antagonists block L-DOPA-induced air-stepping in decerebrate neonatal rats. Brain Res. Dev. Brain Res. 100, 130–132 10.1016/s0165-3806(97)00027-8 9174256

[B90] McDearmid J. R. Scrymgeour-Wedderburn J. F. Sillar K. T. (1997). Aminergic modulation of glycine release in a spinal network controlling swimming in Xenopus laevis. J. Physiol. 503, 111–117 10.1111/j.1469-7793.1997.111bi.x 9288679 PMC1159891

[B158] McEwen M. L. van Hartesveldt C. Stehouwer D. J. (1997). L-DOPA and quipazine elicit air-stepping in neonatal rats with spinal cord transections. Behav. Neurosci. 111, 825–33111, 825–833 10.1037//0735-7044.111.4.825 9267660

[B91] McLean D. L. Sillar K. T. (2004a). Divergent actions of serotonin receptor activation during fictive swimming in frog embryos. J. Comp. Physiol. A Neuroethol. Sens. Neural Behav. Physiol. 190, 391–402 10.1007/s00359-004-0504-9 14991304

[B92] McLean D. L. Sillar K. T. (2004b). Metamodulation of a spinal locomotor network by nitric oxide. J. Neurosci. 24, 9561–9571 10.1523/jneurosci.1817-04.2004 15509743 PMC6730165

[B93] McPherson D. R. Kemnitz C. P. (1994). Modulation of lamprey fictive swimming and motoneuron physiology by dopamine and its immunocytochemical localization in the spinal cord. Neurosci. Lett. 166, 23–26 10.1016/0304-3940(94)90831-1 8190353

[B159] Miles G. B. Sillar K. T. (2011). Neuromodulation of vertebrate locomotor control networks. Physiology (Bethesda, Md) 26, 393–411 10.1152/physiol.00013.2011 22170958

[B94] Missale C. Nash S. R. Robinson S. W. Jaber M. Caron M. G. (1998). Dopamine receptors: from structure to function. Physiol. Rev. 78, 189–225 9457173 10.1152/physrev.1998.78.1.189

[B95] Montague S. J. Fenrich K. K. Mayer-Macaulay C. Maratta R. Neuber-Hess M. S. Rose P. K. (2013). Nonuniform distribution of contacts from noradrenergic and serotonergic boutons on the dendrites of cat splenius motoneurons. J. Comp. Neurol. 521, 638–656 10.1002/cne.23196 22821606

[B96] Moore R. Y. Bloom F. E. (1979). Central catecholamine neuron systems: anatomy and physiology of the norepinephrine and epinephrine systems. Annu. Rev. Neurosci. 2, 113–168 10.1146/annurev.ne.02.030179.000553 231924

[B97] Murray K. C. Nakae A. Stephens M. J. Rank M. D’Amico J. Harvey P. J. (2010). Recovery of motoneuron and locomotor function after spinal cord injury depends on constitutive activity in 5-HT2C receptors. Nat. Med. 16, 694–700 10.1038/nm.2160 20512126 PMC3107820

[B98] Musienko P. van den Brand R. Märzendorfer O. Roy R. R. Gerasimenko Y. Edgerton V. R. (2011). Controlling specific locomotor behaviors through multidimensional monoaminergic modulation of spinal circuitries. J. Neurosci. 31, 9264–9278 10.1523/JNEUROSCI.5796-10.2011 21697376 PMC3422212

[B99] Ochi J. Yamamoto T. Hosoya Y. (1979). Comparative study of the monoamine neuron system in the spinal cord of the lamprey and hagfish. Arch. Histol. Jpn. 42, 327–336 10.1679/aohc1950.42.327 539888

[B100] Oh M. S. Hong S. J. Huh Y. Kim K.-S. (2009). Expression of transgenes in midbrain dopamine neurons using the tyrosine hydroxylase promoter. Gene Ther. 16, 437–440 10.1038/gt.2008.148 18800154 PMC2747767

[B101] Pappas S. S. Behrouz B. Janis K. L. Goudreau J. L. Lookingland K. J. (2008). Lack of D2 receptor mediated regulation of dopamine synthesis in A11 diencephalospinal neurons in male and female mice. Brain Res. 1214, 1–10 10.1016/j.brainres.2008.03.010 18462709

[B102] Pappas S. S. Tiernan C. T. Behrouz B. Jordan C. L. Breedlove S. M. Goudreau J. L. (2010). Neonatal androgen-dependent sex differences in lumbar spinal cord dopamine concentrations and the number of A11 diencephalospinal dopamine neurons. J. Comp. Neurol. 518, 2423–2436 10.1002/cne.22340 20503420 PMC3884812

[B160] Peck J. H. Gaier E. Stevens E. Repicky S. Harris-Warrick R. M. (2006). Amine modulation of Ih in a small neural network. J. Neurophysiol. 96, 2931–2940 10.1152/jn.00423.2005 16943317

[B103] Peyron C. Luppi P. H. Kitahama K. Fort P. Hermann D. M. Jouvet M. (1995). Origin of the dopaminergic innervation of the rat dorsal raphe nucleus. Neuroreport 6, 2527–2531 10.1097/00001756-199512150-00019 8741755

[B104] Pierre J. Mahouche M. Suderevskaya E. I. Reperant J. Ward R. (1997). Immunocytochemical localization of dopamine and its synthetic enzymes in the central nervous system of the lamprey Lampetra fluviatilis. J. Comp. Neurol. 380, 119–135 10.1002/(sici)1096-9861(19970331)380:1<119::aid-cne9>3.3.co;2-j 9073087

[B161] Prinz A. A. Bucher D. Marder E. (2004). Similar network activity from disparate circuit parameters. Nat. Neurosci. 7, 1345–1352 10.1038/nn1352 15558066

[B162] Puhl J. G. Masino M. A. Mesce K. A. (2012). Necessary, sufficient and permissive: a single locomotor command neuron important for intersegmental coordination. J. Neurosci. 32, 17646–17657 10.1523/JNEUROSCI.2249-12.2012 23223287 PMC3538829

[B105] Puhl J. G. Mesce K. A. (2008). Dopamine activates the motor pattern for crawling in the medicinal leech. J. Neurosci. 28, 4192–4200 10.1523/JNEUROSCI.0136-08.2008 18417698 PMC2529178

[B106] Qu S. Ondo W. G. Zhang X. Xie W. J. Pan T. H. Le W. D. (2006). Projections of diencephalic dopamine neurons into the spinal cord in mice. Exp. Brain Res. 168, 152–156 10.1007/s00221-005-0075-1 16044299

[B107] Rajaofetra N. Ridet J. L. Poulat P. Marlier L. Sandillon F. Geffard M. (1992). Immunocytochemical mapping of noradrenergic projections to the rat spinal cord with an antiserum against noradrenaline. J. Neurocytol. 21, 481–494 10.1007/bf01186952 1500947

[B108] Rajaofetra N. Sandillon F. Geffard M. Privat A. (1989). Pre- and post-natal ontogeny of serotonergic projections to the rat spinal cord. J. Neurosci. Res. 22, 305–321 10.1002/jnr.490220311 2709447

[B109] Rauscent A. Einum J. Le Ray D. Simmers J. Combes D. (2009). Opposing aminergic modulation of distinct spinal locomotor circuits and their functional coupling during amphibian metamorphosis. J. Neurosci. 29, 1163–1174 10.1523/JNEUROSCI.5255-08.2009 19176825 PMC6665137

[B110] Reimer M. M. Norris A. Ohnmacht J. Patani R. Zhong Z. Dias T. B. (2013). Dopamine from the brain promotes spinal motor neuron generation during development and adult regeneration. Dev. Cell 25, 478–491 10.1016/j.devcel.2013.04.012 23707737

[B111] Rémy-Néris O. Barbeau H. Daniel O. Boiteau F. Bussel B. (1999). Effects of intrathecal clonidine injection on spinal reflexes and human locomotion in incomplete paraplegic subjects. Exp. Brain Res. 129, 433–440 10.1007/s002210050910 10591914

[B112] Ridet J. L. Sandillon F. Rajaofetra N. Geffard M. Privat A. (1992). Spinal dopaminergic system of the rat: light and electron microscopic study using an antiserum against dopamine, with particular emphasis on synaptic incidence. Brain Res. 598, 233–241 10.1016/0006-8993(92)90188-f 1486484

[B113] Rossignol S. Chau C. Brustein E. Giroux N. Bouyer L. Barbeau H. (1998). Pharmacological activation and modulation of the central pattern generator for locomotion in the cat. Ann. N Y Acad. Sci. 860, 346–359 10.1111/j.1749-6632.1998.tb09061.x 9928324

[B114] Rossignol S. Frigon A. (2011). Recovery of locomotion after spinal cord injury: some facts and mechanisms. Annu. Rev. Neurosci. 34, 413–440 10.1146/annurev-neuro-061010-113746 21469957

[B115] Rowland J. W. Hawryluk G. W. J. Kwon B. Fehlings M. G. (2008). Current status of acute spinal cord injury pathophysiology and emerging therapies: promise on the horizon. Neurosurg. Focus 25:E2 10.3171/FOC.2008.25.11.E2 18980476

[B116] Ryu S. Mahler J. Acampora D. Holzschuh J. Erhardt S. Omodei D. (2007). Orthopedia homeodomain protein is essential for diencephalic dopaminergic neuron development. Curr. Biol. 17, 873–880 10.1016/j.cub.2007.04.003 17481897

[B117] Schmidt B. J. Jordan L. M. (2000). The role of serotonin in reflex modulation and locomotor rhythm production in the mammalian spinal cord. Brain Res. Bull. 53, 689–710 10.1016/s0361-9230(00)00402-0 11165804

[B118] Schotland J. Shupliakov O. Wikström M. A. Brodin L. Srinivasan M. You Z. B. (1995). Control of lamprey locomotor neurons by colocalized monoamine transmitters. Nature 374, 266–268 10.1038/374266a0 7885446

[B163] Shapiro M. G. Frazier S. J. Lester H. A. (2012). Unparalleled control of neural activity using orthogonal pharmacogenetics. ACS Chem. Neurosci. 3, 619–629 10.1021/cn300053q 22896806 PMC3419455

[B166] Sharples S. A. Humphreys J. M. Mayr K. Krajacic A. Dhoopar S. A. Delaloye N. (2013). Dopaminergic contribution to locomotion in the neonatal and adult mouse. (Poster 559.11/ZZ15, Society for Neuroscience, San Diego, California, USA). 21969584

[B119] Shupliakov O. Pieribone V. A. Gad H. Brodin L. (1995). Synaptic vesicle depletion in reticulospinal axons is reduced by 5-hydroxytryptamine: direct evidence for presynaptic modulation of glutamatergic transmission. Eur. J. Neurosci. 7, 1111–1116 10.1111/j.1460-9568.1995.tb01099.x 7613617

[B120] Sickles A. E. Stehouwer D. J. van Hartesveldt C. (1992). Dopamine D1 and D2 antagonists block L-dopa-elicited air-stepping in neonatal rats. Brain Res. Dev. Brain Res. 68, 17–22 10.1016/0165-3806(92)90243-p 1387836

[B121] Sillar K. T. Reith C. A. McDearmid J. R. (1998). Development and aminergic neuromodulation of a spinal locomotor network controlling swimming in Xenopus larvae. Ann. N Y Acad. Sci. 860, 318–332 10.1111/j.1749-6632.1998.tb09059.x 9928322

[B122] Skagerberg G. Lindvall O. (1985). Organization of diencephalic dopamine neurones projecting to the spinal cord in the rat. Brain Res. 342, 340–351 10.1016/0006-8993(85)91134-5 4041835

[B123] Smith D. O. Lowe D. Temkin R. Jensen P. Hatt H. (1995). Dopamine enhances glutamate-activated currents in spinal motoneurons. J. Neurosci. 15, 3905–3912 7538567 10.1523/JNEUROSCI.15-05-03905.1995 PMC6578203

[B124] Sqalli-Houssaini Y. Cazalets J. R. (2000). Noradrenergic control of locomotor networks in the in vitro spinal cord of the neonatal rat. Brain Res. 852, 100–109 10.1016/s0006-8993(99)02219-2 10661501

[B125] Stuart D. G. Hultborn H. (2008). Thomas Graham Brown (1882–1965), Anders Lundberg (1920-) and the neural control of stepping. Brain Res. Rev. 59, 74–95 10.1016/j.brainresrev.2008.06.001 18582502

[B126] Svensson E. Woolley J. Wikström M. A. Grillner S. (2003). Endogenous dopaminergic modulation of the lamprey spinal locomotor network. Brain Res. 970, 1–8 10.1016/s0006-8993(02)04216-6 12706243

[B127] Tamae A. Nakatsuka T. Koga K. Kato G. Furue H. Katafuchi T. (2005). Direct inhibition of substantia gelatinosa neurones in the rat spinal cord by activation of dopamine D2-like receptors. J. Physiol. 568, 243–253 10.1113/jphysiol.2005.091843 15975975 PMC1474768

[B128] Tarazi F. I. Baldessarini R. J. (2000). Comparative postnatal development of dopamine D(1), D(2) and D(4) receptors in rat forebrain. Int. J. Dev. Neurosci. 18, 29–37 10.1016/s0736-5748(99)00108-2 10708903

[B164] Taylor L. L. Sickles A. E. Stehouwer D. J. Van Hartesveldt C. (1994). Noradrenergic alpha-1 and alpha-2 antagonists block L-dopa-induced air-stepping in neonatal rats. Brain Res. Dev. Brain Res. 79, 242–248 10.1016/0165-3806(94)90128-7 7955322

[B129] Thirumalai V. Cline H. T. (2008). Endogenous dopamine suppresses initiation of swimming in prefeeding zebrafish larvae. J. Neurophysiol. 100, 1635–1648 10.1152/jn.90568.2008 18562547 PMC2544474

[B130] Ugrumov M. V. (2009). Non-dopaminergic neurons partly expressing dopaminergic phenotype: distribution in the brain, development and functional significance. J. Chem. Neuroanat. 38, 241–256 10.1016/j.jchemneu.2009.08.004 19698780

[B131] van Dongen P. A. Grillner S. Hokfelt T. (1986). 5-Hydroxytryptamine (serotonin) causes a reduction in the afterhyperpolarization following the action potential in lamprey motoneurons and premotor interneurons. Brain Res. 366, 320–325 10.1016/0006-8993(86)91310-7 3008911

[B165] Vidal-Gadea A. Topper S. Young L. Crisp A. Kressin L. Elbel E. (2011). Caenorhabditis elegans selects distinct crawling and swimming gaits via dopamine and serotonin. Proc. Natl. Acad. Sci. U S A 108, 17504–17509 10.1073/pnas.1108673108 21969584 PMC3198358

[B132] Wallén P. Buchanan J. T. Grillner S. Hill R. H. Christenson J. Hokfelt T. (1989). Effects of 5-hydroxytryptamine on the afterhyperpolarization, spike frequency regulation and oscillatory membrane properties in lamprey spinal cord neurons. J. Neurophysiol. 61, 759–768 2542472 10.1152/jn.1989.61.4.759

[B133] Wang D. Grillner S. Wallén P. (2011). 5-HT and dopamine modulates CaV1.3 calcium channels involved in postinhibitory rebound in the spinal network for locomotion in lamprey. J. Neurophysiol. 105, 1212–1224 10.1152/jn.00324.2009 21228305

[B134] Whelan P. Bonnot A. O’Donovan M. J. (2000). Properties of rhythmic activity generated by the isolated spinal cord of the neonatal mouse. J. Neurophysiol. 84, 2821–2833 11110812 10.1152/jn.2000.84.6.2821

[B135] Wilson J. M. Hartley R. Maxwell D. J. Todd A. J. Lieberam I. Kaltschmidt J. A. (2005). Conditional rhythmicity of ventral spinal interneurons defined by expression of the Hb9 homeodomain protein. J. Neurosci. 25, 5710–5719 10.1523/jneurosci.0274-05.2005 15958737 PMC6724883

[B136] Wolf M. E. Roth R. H. (1990). Autoreceptor regulation of dopamine synthesis. Ann. N Y Acad. Sci. 604, 323–343 10.1111/j.1749-6632.1990.tb32003.x 2171398

[B137] Yoshida M. Tanaka M. (1988). Existence of new dopaminergic terminal plexus in the rat spinal cord: assessment by immunohistochemistry using anti-dopamine serum. Neurosci. Lett. 94, 5–9 10.1016/0304-3940(88)90261-3 3071747

[B138] Zhao H. Zhu W. Pan T. Xie W. Zhang A. Ondo W. G. (2007). Spinal cord dopamine receptor expression and function in mice with 6-OHDA lesion of the A11 nucleus and dietary iron deprivation. J. Neurosci. Res. 85, 1065–1076 10.1002/jnr.21207 17342757

[B139] Zhu H. Clemens S. Sawchuk M. A. Hochman S. (2007). Expression and distribution of all dopamine receptor subtypes (D(1)-D(5)) in the mouse lumbar spinal cord: a real-time polymerase chain reaction and non-autoradiographic in situ hybridization study. Neuroscience 149, 885–897 10.1016/j.neuroscience.2007.07.052 17936519 PMC2185067

